# Simu-dependent clearance of dying cells regulates macrophage function and inflammation resolution

**DOI:** 10.1371/journal.pbio.2006741

**Published:** 2019-05-14

**Authors:** Hannah Grace Roddie, Emma Louise Armitage, Jonathon Alexis Coates, Simon Andrew Johnston, Iwan Robert Evans

**Affiliations:** 1 Department of Infection, Immunity and Cardiovascular Disease, University of Sheffield, Sheffield, United Kingdom; 2 The Bateson Centre, University of Sheffield, Sheffield, United Kingdom; 3 Department of Biomedical Science, University of Sheffield, Sheffield, United Kingdom; National Cancer Institute, United States of America

## Abstract

Macrophages encounter and clear apoptotic cells during normal development and homeostasis, including at numerous sites of pathology. Clearance of apoptotic cells has been intensively studied, but the effects of macrophage–apoptotic cell interactions on macrophage behaviour are poorly understood. Using *Drosophila* embryos, we have exploited the ease of manipulating cell death and apoptotic cell clearance in this model to identify that the loss of the apoptotic cell clearance receptor Six-microns-under (Simu) leads to perturbation of macrophage migration and inflammatory responses via pathological levels of apoptotic cells. Removal of apoptosis ameliorates these phenotypes, while acute induction of apoptosis phenocopies these defects and reveals that phagocytosis of apoptotic cells is not necessary for their anti-inflammatory action. Furthermore, Simu is necessary for clearance of necrotic debris and retention of macrophages at wounds. Thus, Simu is a general detector of damaged self and represents a novel molecular player regulating macrophages during resolution of inflammation.

## Introduction

During development and throughout life, cells are eliminated by programmed cell death and rapidly cleared by phagocytes such as macrophages and glia. Failures in apoptotic cell clearance (efferocytosis) are thought to contribute to disease progression in multiple chronic inflammatory conditions (e.g., chronic obstructive pulmonary disease [COPD]) [1] and autoimmune dysfunction [2], while effective removal of apoptotic cells helps drive resolution of inflammation [3]. Given that macrophage interactions with apoptotic cells can be found in multiple human pathologies, we wished to understand how such interactions affect macrophage behaviour at a cellular level. Therefore, to study the effects of developmental and pathological levels of apoptosis on macrophages, we used *Drosophila* embryos, exploiting the ease of manipulating cell death and apoptotic cell clearance during in vivo imaging in this model.

*Drosophila* embryos contain a population of highly motile, macrophage-like cells (plasmatocytes, one of the three hemocyte cell types) that disperse to cover the entire embryo during development, removing apoptotic cells and secreting extracellular matrix as they migrate [4]. In addition to their functional similarities with vertebrate white blood cells, fly blood cells share genetic specification via the action of GATA and Runx family members [5]. Dispersal of embryonic macrophages is controlled through expression of platelet-derived growth factor/vascular endothelial growth factor (PDGF/VEGF) related ligands (Pvfs) along their routes [6–8], coupled with physical constraints [9]. Cell–cell repulsion [10] and down-regulation of Pvfs [8] contribute to the timing and stereotyped nature of later migratory events—lateral migration of macrophages from the ventral midline to the edges of the developing ventral nerve cord (VNC).

*Drosophila* macrophages prioritise their activites in the developing embryo, with apoptotic cell clearance taking precedence over their dispersal [11]. Surprisingly, prior exposure to apoptotic cells seems required for normal responses to injury and infection [12]. Inflammatory responses to sterile injuries strongly resemble those observed in vertebrates with a Duox (also known as Cy) dependent burst in hydrogen peroxide essential for normal recruitment to sites of damage [13,14] and a genetic requirement for specific Src family kinases within innate immune cells [15,16].

During embryonic development, *Drosophila* macrophages work in concert with the glia of the central nervous system (CNS) to remove apoptotic cells [17]. Both phagocytes use a variety of receptors to recognise and engulf apoptotic cells [18], one of which is Six-microns-under (Simu), also known as Nimrod C4. Simu is a member of the Nimrod family of cell surface receptors with homology to members of the cell death abnormality gene 1 (CED-1) family of receptors, e.g., CED-1 (*Caenorhabditis elegans*) [19], Draper (*Drosophila melanogaster*) [20], Jedi-1, and multiple epithelial growth factor-like domains 10 (MEGF10, mouse) [21]. Simu binds phosphatidylserine (PS) using its elastin microfibril interface-located protein (EMILIN) like and NIM1 and NIM2 domains at the N-terminus [22], and its absence from macrophages and glia leads to a failure in removing developmentally programmed apoptosis [23]. In some contexts, Simu may operate upstream of Draper [23], though direct physical interaction has not been demonstrated.

Here, we used *simu* mutant fly embryos to challenge macrophages with pathological levels of apoptotic cell death to address how this affected their subsequent behaviour. We show that apoptotic cell death contributes to developmental dispersal of macrophages and that excessive amounts of apoptosis also induce defects in dispersal and migration. Chronic levels of uncleared apoptotic cells and acute induction of apoptotic cell death impair wound responses in vivo, importantly, without a requirement for phagocytosis of these dying cells. Finally, in our new paradigm, we demonstrate a novel role for Simu in retention of macrophages at sites of necrotic wounds and reveal that Simu facilitates clearance of nonapoptotic cells at such sites of injury. Therefore, the role of Simu is not limited to clearance of apopotic cells but regulates responses to damaged self in general.

## Results

### Apoptotic cells regulate developmental dispersal of macrophages

**Fig 1:**
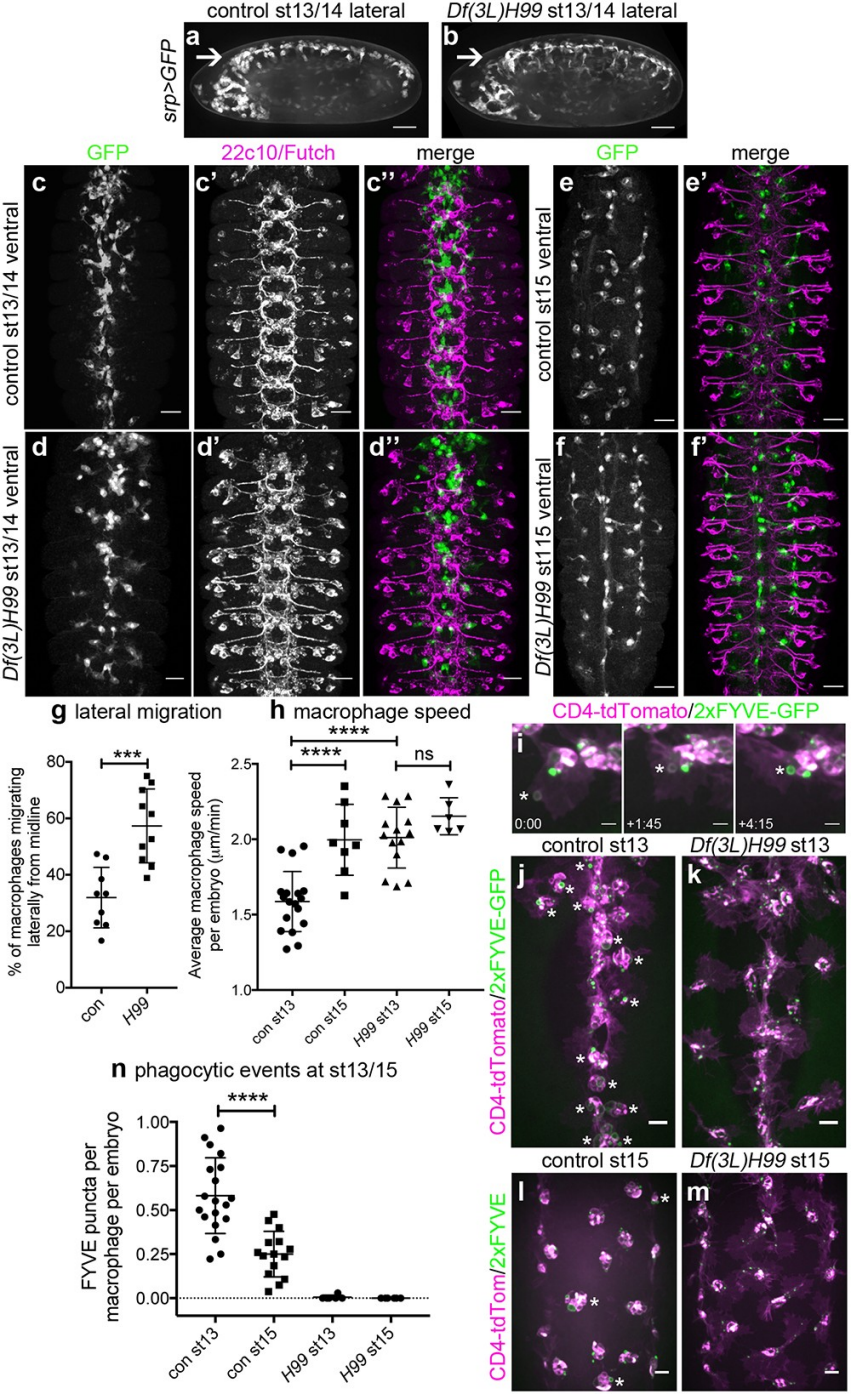
Apoptotic cell death contributes to developmental dispersal of *Drosophila* embryonic macrophages. (a–b) Lateral projections (anterior is left) of control (*w;srp-GAL4*,*UAS-GFP*) and apoptosis-null *Df(3L)H99* mutant embryos (*w;srp-GAL4*,*UAS-GFP;Df(3L)H99*) showing macrophage distribution at stage 13/14 of development, including along ventral midline (arrow). (c–d) Ventral projections (anterior is up) showing macrophages (anti-GFP staining, green in merge) and structure of the CNS (22c10/anti-Futch staining, purple in merge) at stage 13 of development in controls (*w;;crq-GAL4*,*UAS-GFP*) and in the absence of apoptosis (*w;;Df(3L)H99*,*crq-GAL4*,*UAS-GFP*). Macrophage projections constructed from z-slices corresponding to superficial macrophages on the ventral side of the VNC only, whereas Futch projection covers the entire volume of the VNC. (e–f) Ventral projections (anterior is up) showing macrophage distribution (GFP) alone (e–f), or merged with Futch staining (e’–f’) to show CNS structure in control and *Df(3L)H99* mutant embryos at stage 15 (genotypes as per c–d). (g) Scatterplot showing percentage of macrophages on the midline that move laterally at stage 13 of development in control and apoptosis-null embryos (*n* = 9 and 10, respectively; *P* = 0.0006, Mann–Whitney test); lateral migration concludes around the end of stage 14. (h) Scatterplot showing speed per macrophage, per embryo (μm per min) in control and apoptosis-null embryos at stage 13 (*P* < 0.0001) and stage 15 (*n* = 18, 8, 14, 6 [left–right]; *P* = 0.47, one-way ANOVA with Tukey’s multiple comparison test). Genotypes in (g–h) are as per (c–f). (i) Stills from movie of macrophages phagocytosing at stage 13 on the ventral midline in a control embryo (*w;srp-GAL4*,*UAS-2xFYVE-GFP;crq-GAL4*,*UAS-CD4-tdTomato*). GFP and tdTomato are shown in green and purple, respectively, while asterisk shows nascent phagosome at indicated times (mins:secs) as it becomes positive for the PI3P sensor 2xFYVE-GFP post engulfment; CD4-tdTomato labels membranes. (j–m) Ventral projections of control (*w;srp-GAL4*,*UAS-2xFYVE-GFP;crq-GAL4*,*UAS-tdTomato*) and apoptosis-null embryos (*w;srp-GAL4*,*UAS-2xFYVE-GFP;crq-GAL4*,*UAS-tdTomato;Df(3L)H99*) at stage 13 (j–k) and stage 15 (l–m), showing recent phagocytic events via 2xFYVE-GFP sensor for PI3P. Asterisks show examples of 2xFYVE-GFP positive cells (j, l). (n) Scatterplot showing number of phagocytic events (via 2xFYVE-GFP positive vacuoles) per macrophage, per embryo at stages 13 and 15 (*n* = 19 and 15, respectively; *P* < 0.0001, Mann–Whitney test). Lines and error bars show mean and standard deviation (g–h, n); *** and **** denote *P* < 0.001 and *P* < 0.0001; scale bars represent 50 μm (a–b), 10 μm (c–f), 5 μm (i), and 10 μm (j–m). All data used to plot graphs may be found in Supporting information file S1 Data. CNS, central nervous system; GFP, green fluorescent protein; PI3P, phosphatidylinositol 3-phosphate; UAS, upstream activating sequence.

To understand the role of developmentally programmed apoptosis in dispersal of macrophages, *Df(3L)H99* mutant embryos, which lack all apoptosis owing to deletion of the proapoptotic genes *hid*, *reaper*, and *grim* [24], were examined. As per previous reports [6,25], macrophage dispersal was grossly normal in the absence of apoptosis (Fig 1a and 1b). However, detailed analysis at stage 13 of development revealed that more macrophages were present in lateral positions in embryos that lacked apoptosis compared to controls (Fig 1c and 1d). By stage 15, macrophage localisation on the VNC was similar in controls and embryos lacking apoptosis (Fig 1e and 1f). Macrophage dispersal and VNC development are interdependent [9], but in this instance, we saw no obvious VNC defects in the absence of apoptotic cell death [26], making this dispersal phenotype unlikely to be a consequence of morphogenetic defects (Fig 1e and 1f). Consistent with this, live imaging of GFP-labelled macrophages at stage 13 revealed that significantly greater numbers of macrophages left the ventral midline and migrated laterally in the absence of apoptosis (Fig 1g). Furthermore, this precocious migration was reflected in an enhancement in macrophage speeds in the absence of apoptosis at stage 13, while speeds were similar to controls at stage 15 (Fig 1h).

By stage 15 of development, the majority of apoptosis occurs within the CNS [26], and the formation of septate junctions by surface glia prevents macrophages from accessing these dying cells [27]. Therefore, we predicted that macrophages at stage 15 would have fewer apoptotic cell phagosomes in comparison to stage 13. To test this, a phosphatidylinositol 3-phosphate (PI3P) sensor was expressed in macrophages (2xFYVE-GFP) [28] to mark nascent phagosomes [29] (Fig 1i). Consistent with sequestration of apoptosis within the CNS, there were significantly fewer nascent phagosomes visible within macrophages at stage 15 compared to stage 13 (Fig 1j, 1l and 1n), and such phagosomes were absent in apoptosis-null embryos (Fig 1k, 1m and 1n). Therefore, it is only once macrophages become less exposed to apoptotic cell death that they become more motile, suggesting that interactions with apoptotic cells restrict macrophages from migrating laterally. Taken together, these data suggest that interactions between developmentally programmed apoptotic cell death and macrophages can delay lateral migration and contribute to the regulation of macrophage dispersal in *Drosophila* embryos.

### Pathological levels of apoptosis are associated with developmental defects in macrophage dispersal

**Fig 2:**
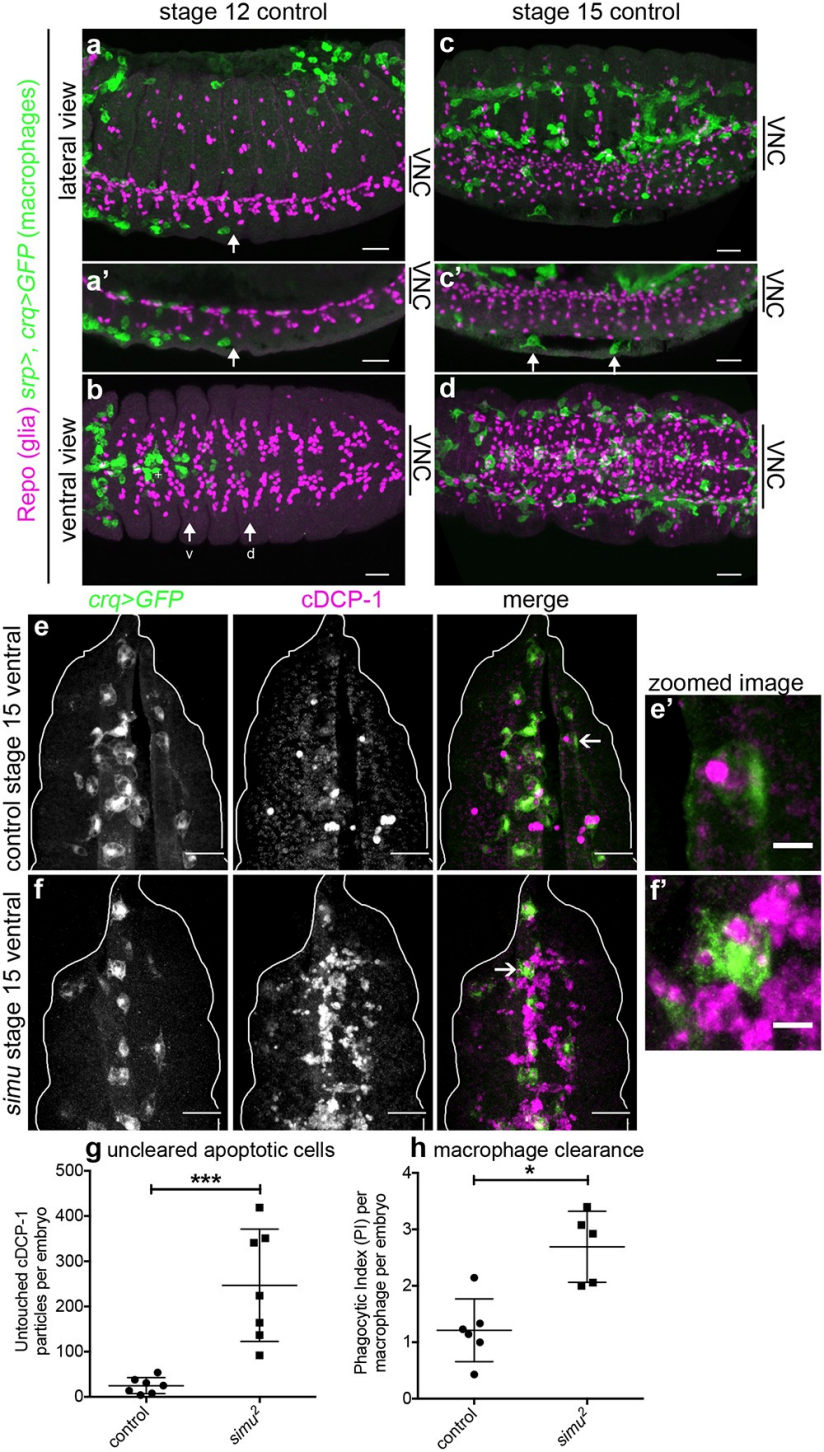
Loss of *simu* function results in exposure of macrophages to pathological levels of uncleared apoptotic cells. (a–d) Maximum projections of control embryos (*w;srp-GAL4*,*UAS-GFP/+;crq-GAL4*,*UAS-GFP/+*) immunostained to show close contact of macrophages (anti-GFP, green), and glia (anti-Repo, purple) at stage 12 during migration along both sides of the ventral midline (a, lateral; b, ventral) and later in development at stage 15 (c, lateral; d, ventral). (a’) and (c’) are maximum projections of a smaller number of z-slices to show position of macrophages between epidermis and and VNC. Anterior is left and arrows show pioneer macrophages; arrows ‘v’ and ‘d’ in (b) label most advanced macrophages moving along the ventral and dorsal side of the VNC, respectively; arrows in (c’) show two macrophages between VNC and epidermis. (e–f) Maximum projections of stage 15 control and *simu* mutant embryos showing macrophages (GFP, green in merge) and apoptotic particles (cDCP-1, purple in merge), respectively. Projections are ventral views and correspond to void between epidermis and VNC. (e’–f’) Show zooms of macrophages indicated by arrows in (e–f); white line shows edge of embryos. (g) Scatterplot of untouched apoptotic punctae (cDCP-1 punctae not in contact with macrophages in the field of view) per embryo in control and *simu* mutant embryos at stage 15 (*n* = 7 per genotype; *P* = 0.0006, Mann–Whitney test). (h) Scatterplot of phagocytic index (cDCP-1 punctae engulfed per macrophage, per embryo) in control and *simu* mutant embryos at stage 15 (*n* = 6 and 5 embryos, respectively; >10 cells analysed per embryo, 96 and 64 macrophages analysed in total, respectively; *P* = 0.017, Mann–Whitney test). Genotypes in (e–h) are *w;;crq-GAL4*,*UAS-GFP* (control) and *w;simu*^*2*^;*crq-GAL4*,*UAS-GFP* (*simu* mutants). Error bars represent mean ± standard deviation; * and *** denote *P* < 0.05 and *P* < 0.001; scale bars represent 10 μm (a–d), 20 μm (e–f), and 5 μm in zoomed panels (e’–f’). All data used to plot graphs may be found in Supporting information file S1 Data. cDCP-1, cleaved death caspase 1; GFP, green fluorescent protein; UAS, upstream activating sequence; VNC, ventral nerve cord.

Having identified a novel role for apoptotic cell death in regulating dispersal of *Drosophila* embryonic macrophages, we wished to investigate the consequences of pathological levels of apoptosis on macrophage behaviour in vivo. Embryonic macrophages migrate along the ventral midline in a constrained channel [9], in close contact with the epithelium and developing CNS (Fig 2a and 2b). Later in development the environment becomes less constricted and macrophages disperse over the ventral side of the embryo, albeit still sandwiched between the epithelium and CNS (Fig 2c and 2d). Thus, macrophages migrate in very close contact with the other main phagocyte population in the developing embryo (the glia of the VNC).

We predicted that removing the apoptotic cell clearance receptor Simu from macrophages and glia would lead to an overstimulation of macrophages with apoptotic cells, owing to a reduction in apoptotic cell clearance by these neighbouring cell populations. To confirm this prediction, apoptotic cell death was visualised by staining embryos using an antibody to cleaved death caspase 1 (cDCP-1), an effector caspase, itself cleaved during apoptosis [30]. Consistent with the role of Simu in apoptotic cell clearance, the absence of *simu* led to large numbers of apoptotic cells remaining unengulfed at stage 15 in the space between the epithelium and developing VNC, in contrast to controls in which very few uncleared apoptotic cells persisted (Fig 2e and 2f). Significantly, *simu* mutants exhibited a large increase in the number of untouched apoptotic cells in this region of the developing embryo (Fig 2g), with only a small increase in the phagocytic index of macrophages (Fig 2h). Untouched apoptotic cell punctae were used as a more conservative estimate of uncleared apoptotic cells, since macrophages were so overwhelmed that it was difficult to discern accurately whether phagocytosis had taken place from images of immunostained embryos. Therefore, in line with previous data [23], we could demonstrate a role for *simu* in apoptotic cell clearance, with the absence of *simu* function leading to pathological levels of apoptosis surrounding macrophages on the ventral side of the developing *Drosophila* embryo.

**Fig 3:**
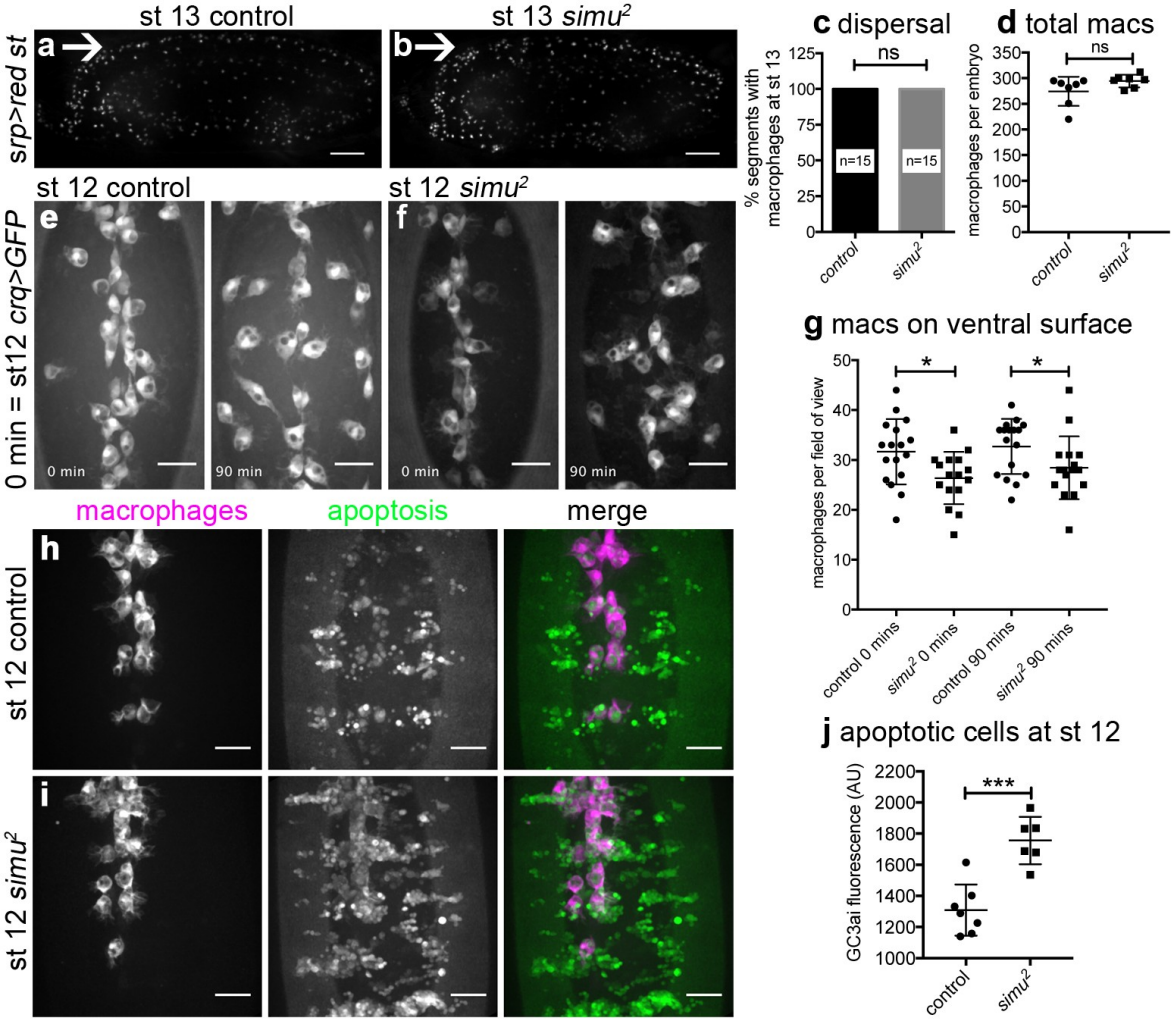
Loss of *simu* function leads to defects in developmental dispersal of macrophages and an early build up of apoptotic cells. (a–b) Lateral images of control (*w;srp-GAL4*,*UAS-red stinger*) and *simu* mutant embryos (*w;simu*^*2*^,*srp-GAL4*,*UAS-red stinger*) showing migration of red stinger-labelled macrophages along the ventral midline at stage 13; arrows indicate ventral midline, anterior is left, ventral is up. (c) Bar graph showing percentage of segments with GFP-labelled macrophages on the ventral side of the VNC at stage 13 in controls (*w;;crq-GAL4*,*UAS-GFP*) and *simu* mutants (*w;simu*^*2*^;*crq-GAL4*,*UAS-GFP*) (*n* = 15 and 15, respectively; *P* > 0.999, Mann–Whitney test). (d) Total numbers of red stinger-labelled macrophages in lateral views of control and *simu* mutants (genotypes as per [a–b] at stage 13; *n* = 7 for each; *P* = 0.066, Mann–Whitney test). (e–f) GFP-labelled macrophages on the ventral side of the embryo in controls (e) and *simu* mutants (f); imaging started at the end of stage 12 with time indicating duration of imaging. (g) Numbers of GFP-labelled macrophages on the ventral surface of embryos at the indicated timepoints in control and *simu* mutants (*n* = 17 and16, respectively, *P* = 0.0162 and 0.046 and at 0 and 90 mins, respectively, Student *t* tests); genotypes as per (c) in e–g. (h–i) Ventral views showing migration of mCherry-labelled macrophages (purple in merge) down the ventral midline (anterior is up) encountering GC3ai-positive apoptotic cells (green in merge) during stage 12 in control and *simu* mutant embryos. (j) GC3ai fluorescence levels (arbitrary units) quantified from average projections of the ventral midline region of control and *simu* mutant embryos at stage 12; genotypes are *w;;da-GAL4*,*UAS-GC3ai/srp-3x-mCherry* (control) and *w;simu*^*2*^;*da-GAL4*,*UAS-GC3ai/srp-3x-mCherry* (*simu*) in (h–j) (*n* = 7 and 6, respectively; *P* = 0.0004, Student *t* test). Lines/bars and error bars show mean and standard deviation; ns, * and *** denote not significant, *P* < 0.05 and *P* < 0.001, respectively; scale bars represent 50 μm (a–b) and 20 μm (e–f, h–i). All data used to plot graphs may be found in Supporting information file S1 Data. GFP, green fluorescent protein; UAS, upstream activating sequence.

After establishing that loss of *simu* function leads to pathological levels of apoptosis in vivo, we sought to test the effect this exerted on macrophage behaviour. Migration of macrophages over the embryo is critical for embryonic development, since removal of dying cells and secretion of matrix are important aspects of morphogenesis [31,32]. *Drosophila* macrophages clear apoptotic cells as they disperse throughout the embryo, contemporaneously with induction of apoptosis [33]. To assess developmental dispersal we scored macrophage progression along the midline in control and *simu* mutant embryos, observing no obvious defects at stage 13 (Fig 3a–3c), nor was there a difference in the total numbers of macrophages in these embryos (Fig 3d), nor a delay in development (S1a Fig). However, quantification of numbers of macrophages on the ventral midline at stage 12 revealed defects in macrophage dispersal in *simu* mutants (Fig 3e–3g; S1 Movie). Live imaging of macrophages from stage 12 onward showed this dispersal defect persisted through development (Fig 3g and S1d Fig) and was associated with a reduction in migration speed (S1b Fig). Macrophage apoptosis did not contribute to this phenotype, with no macrophages adopting morphologies typical of cells undergoing apoptosis in *simu* mutant embryos (16 movies analysed; S1 Movie). Imaging of whole embryos revealed that migration to other regions appeared grossly normal in *simu* mutants (Fig 3a and 3b; S2 Movie).

This suggested that the presence of excessive numbers of apoptotic cells can perturb dispersal of macrophages, consistent with the sensitivity of these cells to developmentally programmed apoptosis (Fig 1c–1h). To image apoptosis and macrophage dispersal simultaneously, we expressed a caspase-activated GFP variant (GC3ai) [34] ubiquitously within embryos; GC3ai marks cells undergoing apoptosis that fragment and are engulfed by macrophages (S1c Fig; S3 Movie). Even at stage 12, there is a significant increase in uncleared apoptotic cells in ventral regions in *simu* mutants (Fig 3h–3j), and macrophages interact with these clusters of dying cells, reducing their dispersal compared to controls (S3 Movie). Thus, in the absence of apoptosis, macrophage dispersal is accelerated (Fig 1c–1h), whereas the presence of large amounts of apoptotic cell death is associated with a restriction in this process. This indicates that apoptotic cells regulate macrophage migration and have the potential to hinder normal development since dispersal is a prerequisite for normal morphogenesis [31,32,35].

### Pathological levels of apoptosis impair basal migration of macrophages

**Fig 4:**
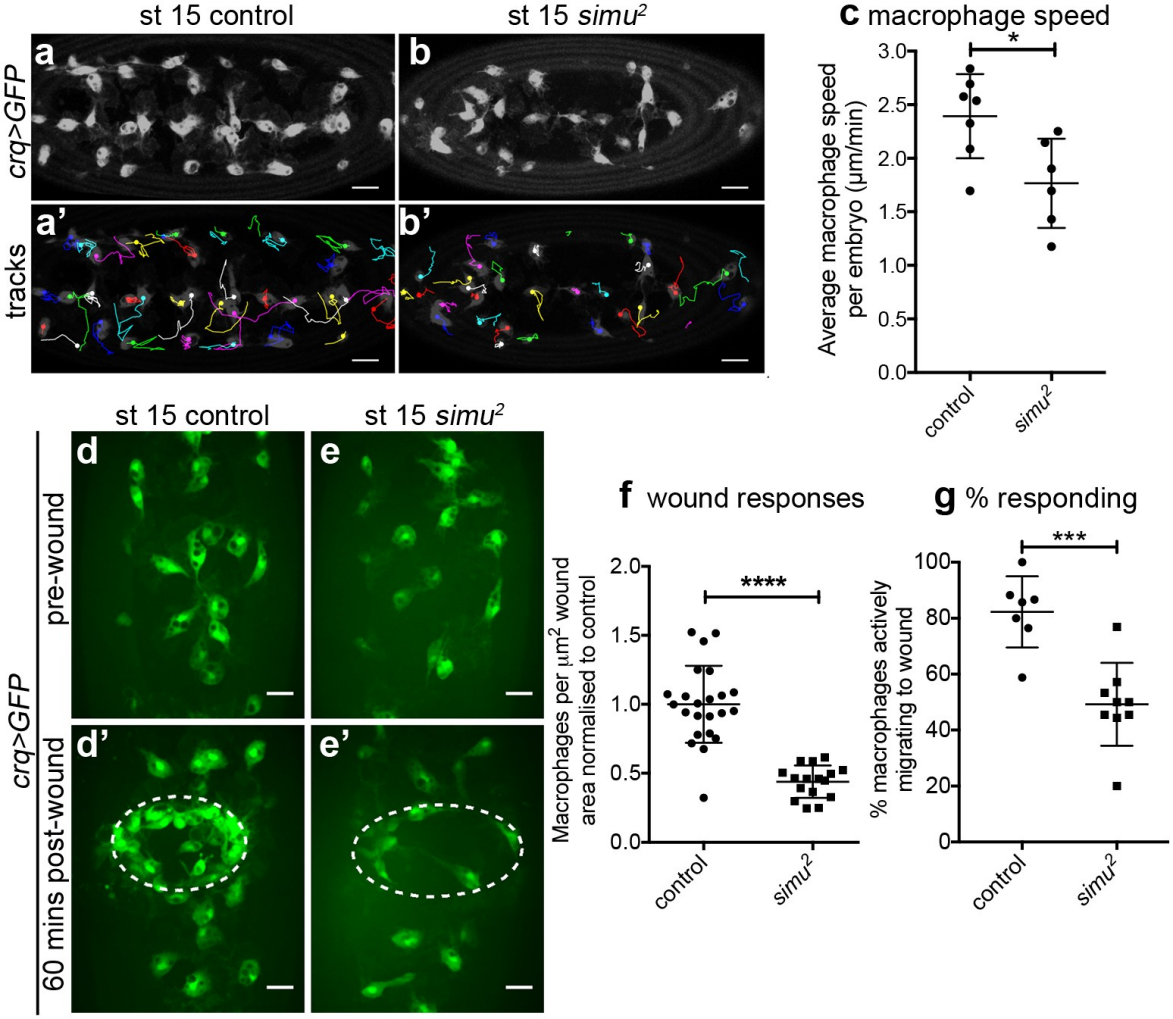
Pathological levels of apoptosis impair motility of macrophages and inflammatory migration to wounds. (a–b) Images of GFP-labelled macrophages and their associated tracks on the ventral midline at stage 15 in control (*w;;crq-GAL4*,*UAS-GFP*, a) and *simu* mutant (*w;simu*^*2*^;*crq-GAL4*,*UAS-GFP*, b) embryos. (a’–b’) Tracks show macrophage migration over a 30-minute period; dots show starting positions of those macrophages present in the first frame of the movie; anterior is left. (c) Scatterplot of average speed per macrophage, per embryo on the ventral midline at stage 15 in μm per min in controls and *simu* mutants (*n* = 7 and 6, respectively; *P* = 0.035, Mann–Whitney test). (d–e) Images of GFP-labelled macrophages on the ventral midline in stage 15 control (d–d’) and *simu* mutant (e–e’) embryos before (d–e) and at 60-minutes post wounding (d’–e’). Dotted lines indicate wound sites. (f) Scatterplot of wound response at 60 minutes (number of macrophages at wound divided by wound area, normalised to control average) in control and *simu* mutant embryos (*n* = 23 and 16, respectively; *P* < 0.0001, Mann–Whitney test). (g) Scatterplot showing percentage of macrophages responding to wounds (percentage of those in field of view at t = 0 not initially in contact with the wound site that reach the wound site in a 60-minute movie) in control and *simu* mutant embryos (*n* = 7 and 9, respectively; *P* = 0.0006, Mann–Whitney test). All genotypes as per (a–b). Lines and error bars show mean and standard deviation; *, ***, and **** denote *P* < 0.05, *P* < 0.001, and *P* < 0.0001 (c, f, g); scale bars represent 20 μm (a–b) and 10 μm (d–e). All data used to plot graphs may be found in Supporting information file S1 Data. GFP, green fluorescent protein; UAS, upstream activating sequence.

Given the impairment in developmental dispersal in the presence of excessive amounts of apoptosis (Fig 3e–3g), we addressed whether macrophage migration was perturbed more generally in *simu* mutants. We measured basal cell motility by imaging the wandering migration of GFP-labelled macrophages at stage 15, finding that migration speeds were significantly reduced in *simu* mutants compared to controls (Fig 4a–4c; S4 Movie). Despite the large numbers of uncleared apoptotic cells surrounding macrophages in *simu* mutants (Fig 2e–2g), there was only a minor stimulation of phagocytosis in *simu* mutants (S2a–S2c Fig), consistent with the role of Simu in apoptotic cell clearance [23]. We were unable to detect obvious or repeated attempts to phagocytose apoptotic cells, suggesting that the defects in migration may not simply be a consequence of frustrated phagocytic events. Furthermore, macrophages exhibited a similar morphology to controls in *simu* mutants, exhibiting large, well-spread lamellipodia, with the only morphological difference detected being decreased circularity (S2a, S2b, S2d and S2e Fig). Therefore, the motility machinery of these embryonic macrophages remains intact, consistent with relatively mild defects in their dispersal (Fig 3). As both basal migration and developmental dispersal were impaired in *simu* mutants, we next turned to an assay of inflammatory migration to address whether other pathologically relevant macrophage behaviours were perturbed in the face of large amounts of uncleared apoptotic cells.

### Pathological levels of apoptosis impair inflammatory migration to wounds

Macrophages undergo a rapid and highly directional inflammatory migration to laser-induced wounds in *Drosophila* embryos [36]. Laser wounds induce calcium waves in the epithelium, leading to production of hydrogen peroxide, which is essential for efficient recruitment of macrophages to these sites of damage [14]. Imaging GFP-labelled macrophages in *simu* mutant embryos revealed a significant defect in recruitment of macrophages to wounds (Fig 4d–4f; S5 Movie). This effect was far greater than the small reduction in macrophage numbers present on the midline ahead of wounding in *simu* mutants (Fig 3e–3g and S1d Fig), suggesting the defect in inflammatory migration is not purely due to a reduction in cells available locally to respond. Furthermore, the proportion of macrophages responding to wounds was also quantified, revealing that a significantly greater percentage of macrophages migrated to wounds in controls compared to *simu* mutants (Fig 4g). Therefore, the ability to respond to wounds is compromised in *simu* mutants at a cellular level, potentially due to the presence of large numbers of uncleared apoptotic cells in the embryonic milieu. Imaging calcium responses following wounding using the cytoplasmic calcium sensor GCamP6M [37] indicated that this reduction in inflammatory recruitment was not due to defects in the generation of wound cues (S2f–S2i Fig).

Wound recruitment defects are specifically associated with loss of *simu* function, since placing the loss-of-function allele *simu*^*2*^ in trans to a deficiency that deletes *simu* also perturbed inflammatory responses (S2j and S2k Fig). Furthermore, re-expression of wild-type *simu* in macrophages ameliorated *simu* mutant defects (S2l and S2m Fig), in line with a role for *simu* in both macrophage and glial-mediated apoptotic cell clearance [23].

### Acute induction of apoptosis is sufficient to impair inflammatory responses

**Fig 5:**
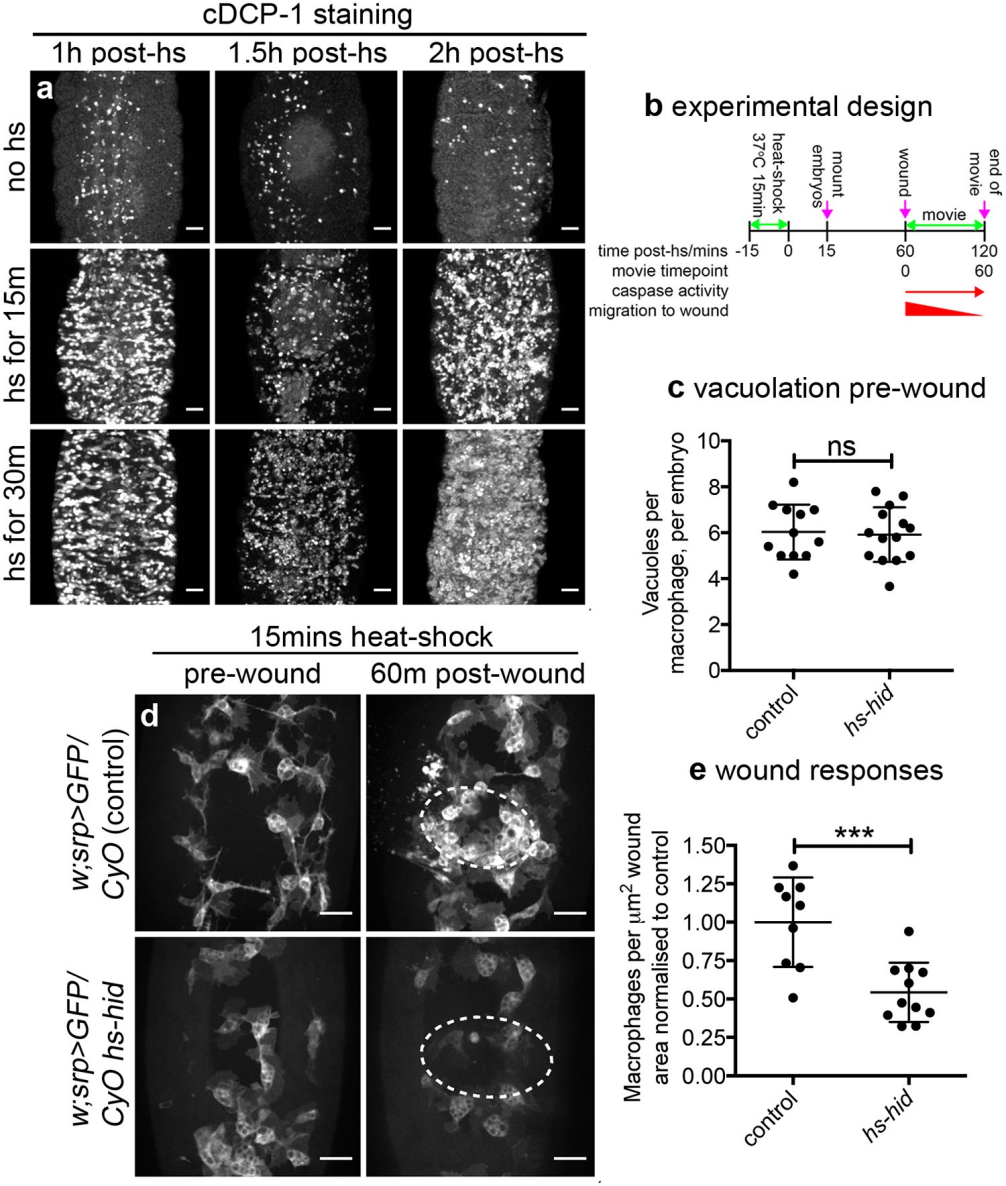
Acute induction of apoptosis impairs inflammatory responses without the requirement for engulfment by macrophages. (a) Ventral projections of embryos containing *hs-hid* (*w;CyO hs-hid/+*) stained for active caspases (anti-cDCP-1) following heat-shock for 0, 15, or 30 minutes; embryos fixed and stained at indicated times. (b) Schematic showing experimental design to induce exogenous apoptosis ahead of clearance by phagocytes in the embryo. (c) Scatterplot showing vacuoles per macrophage, per embryo as a read out of phagocytosis at 60 minutes post heat shock (immediately before wounding) in control and *hs-hid* embryos (*n* = 12 and 14, respectively; *P* = 0.829, Mann–Whitney test). (d) Prewound and 60-minute postwound ventral views of control (*w;srp-GAL4*,*UAS-GFP/CyO*) and *hs-hid* embryos (*w;srp-GAL4*,*UAS-GFP/CyO hs-hid*) subjected to 15-minute heat-shock; experimental design was as per (b), with wounding taking place at 60 minutes post heat shock. Dotted lines show wound edges. (e) Scatterplot of wound responses (density of macrophages at wound sites normalised to wound area and to control average) at 60 minutes post wounding of control and *hs-hid* embryos (*n* = 9 and 11, respectively; *P* = 0.0005, Mann–Whitney test). Line and error bars show mean and standard deviation in all scatterplots; ns and *** denote not significant and *P* < 0.001; scale bars represent 20 μm. All data used to plot graphs may be found in Supporting information file S1 Data. cDCP-1, cleaved death caspase 1; GFP, green fluorescent protein; UAS, upstream activating sequence.

Since pathological levels of apoptosis appeared to impair wound responses in *simu* mutants, we wished to understand whether chronic or acute exposure to dying cells underlied this phenotype. Furthermore, carefully controlled introduction of apoptotic cell death would enable an understanding of whether efferocytosis was necessary for apoptosis-induced impairment of wound responses. To achieve this, the proapoptotic regulator *hid* was overexpressed through a short heat-shock treatment of developing embryos that contained this gene under the control of the *hsp70* promoter (*hs-hid*) [38]. Induction of apoptosis was confirmed by staining for activated caspases (Fig 5a). The length of heat-shock treatment was determined by imaging embryos containing ubiquitous expression of a caspase activity reporter (apoliner) [39] and the *hs-hid* transgene (S3a–S3d Fig; S6 Movie).

Stage 15 embryos were heat-shocked for 15 minutes and immediately mounted for wounding. Wounding was performed 60 minutes after heat shock, a timepoint when caspase activity could be seen in those cells destined to die by apoptosis but ahead of their delamination and engulfment by macrophages (S3a–S3d Fig). The majority of cells that undergo apoptosis appear to be superficial epithelial cells, and these typically remain in the epithelium over the course of our 60-minute wounding experiments (60–120 minutes post heat shock), with a wave of delamination eliminating them from the epithelium by 4.5 hours (±0.3 h standard deviation, *n* = 9 movies; S6 Movie). Calibrating the assay in this fashion meant it was possible to discern whether engulfment was a prerequisite for antagonism of inflammatory responses by apoptotic cell death (Fig 5b). The lack of engulfment at this time point was confirmed via quantification of apoptotic cell–containing vacuoles in macrophages, which are present in the same numbers in macrophages that have been heat shocked in the presence or absence of *hs-hid* (Fig 5c).

Imaging inflammatory responses of macrophages following induction of exogenous apoptosis revealed a significant defect in macrophage recruitment to wounds at 60 minutes post wounding compared to control embryos (Fig 5d and 5e; S7 Movie). Importantly, no macrophages were observed to undergo apoptosis in these assays (S7 Movie), suggesting these cells are somewhat tolerant to *hid* expression, nor is there a reduction in numbers of macrophages ahead of wounding (S3e Fig). Wounding the same embryos in the absence of a heat shock failed to reveal a defect in wound responses (S3f and S3g Fig), indicating impaired wound responses were specific to the induction of exogenous apoptosis and could not have been caused by differences in genetic background (e.g., insertion site of *hs-hid*). Taken together, this suggests that acute induction of apoptosis can attenuate wound responses, phenocopying *simu* mutant embryos in which macrophages are overwhelmed by uncleared apoptotic cells. Furthermore, since we could not detect an increase in phagocytosis at the time of wounding (Fig 5c), this implies that phagocytosis of apoptotic cells is not necessary for these phenotypes.

### Removal of apoptosis from *simu* mutants restores normal migration and improves macrophage responses to wounding

**Fig 6:**
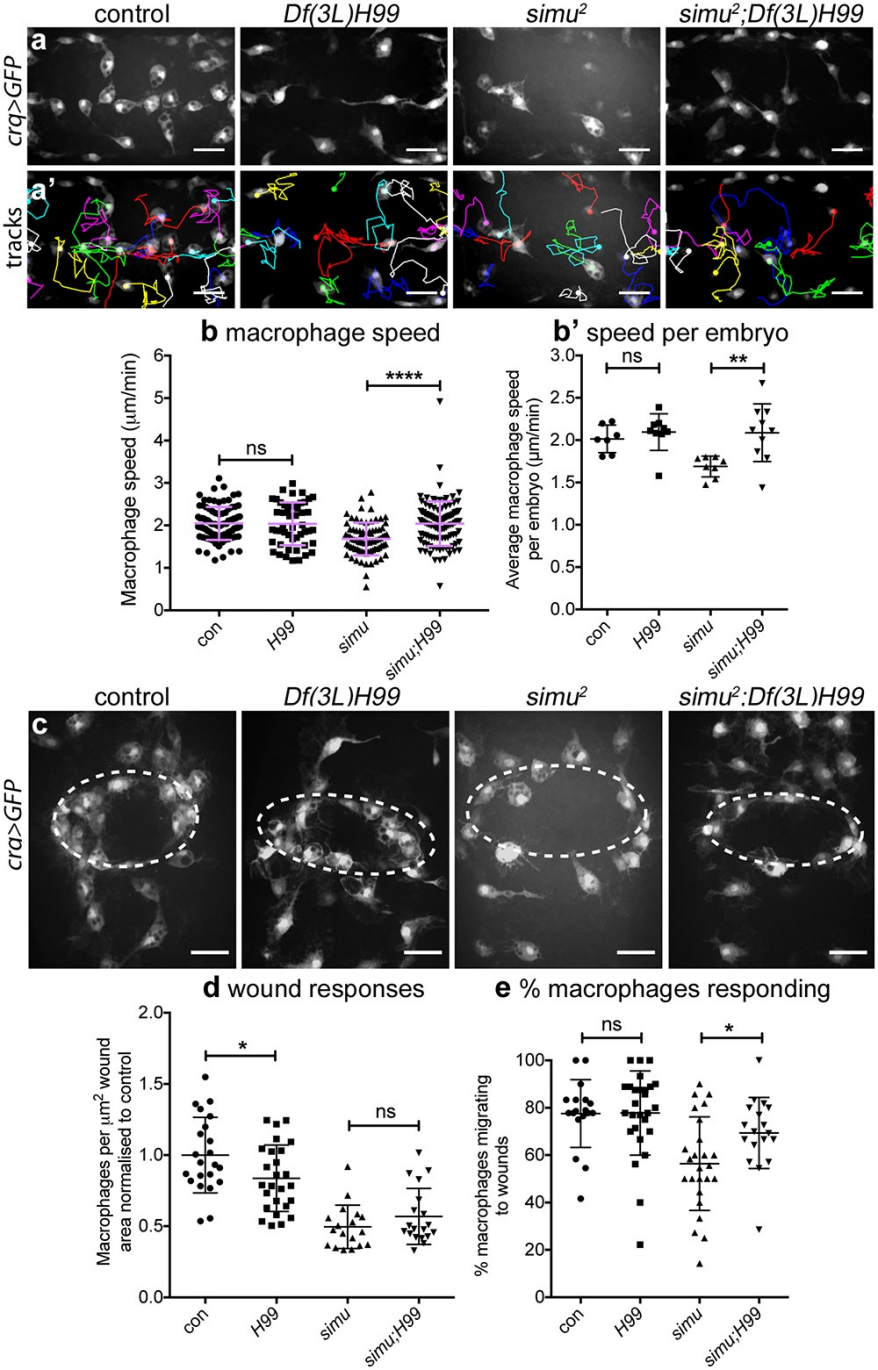
Removal of apoptosis from *simu* mutants rescues wandering migration and initial responses to wounds. (a–a’) Maximum projections of GFP-labelled macrophages on the ventral midline at stage 15 (a) and tracks of their migration in the subsequent 60 minutes (a’) in controls, apoptosis-null embryos (*Df(3L)H99*), *simu* mutants (*simu*^*2*^) and *simu* mutants that lack apoptosis (*simu*^*2*^;*Df(3L)H99*). (b–b’) Scatterplots of speed per macrophage (b) and average speed per embryo (b’) in μm per min at stage 15 in control and apoptosis-null embryos (*n* = 7 and 9, respectively; *P* = 0.252, Mann–Whitney test), and *simu* mutants and *simu* mutants that lack apoptosis (*n* = 8 and 10, respectively; *P* = 0.006, Mann–Whitney test, b’). (c) Maximum projections of macrophages at wounds 60-minutes post wounding in indicated embryos; dotted white ellipses show wound edges. (d) Scatterplot of wound responses (macrophage density at wounds normalised to control average) comparing control and apoptosis-null embryos (*n* = 22 and 26, respectively; *P* = 0.039, Mann–Whitney test), and *simu* mutants and *simu* mutants that lack apoptosis (*n* = 18 and 19, respectively; *P* = 0.30, Mann–Whitney test). (e) Scatterplot of percentage of responding macrophages (percentage that migrate to the wound that were not at the wound at *t* = 0 minutes) comparing control and apoptosis-null embryos (*n* = 18 and 27, respectively; *P* = 0.80, Mann–Whitney test), and *simu* mutants and *simu* mutants that lack apoptosis (*n* = 25 and 18, respectively; *P* = 0.019, Mann–Whitney test). Genotypes for all embryos: control (*w;;crq-GAL4*,*UAS-GFP*), *Df(3L)H99* (*w;;Df(3L)H99*,*crq-GAL4*,*UAS-GFP*), *simu*^*2*^ (*w;simu*^*2*^;*crq-GAL4*,*UAS-GFP*) and *simu*^*2*^;*Df(3L)H99* (*w;simu*^*2*^;*Df(3L)H99*,*crq-GAL4*,*UAS-GFP*). Lines and error bars in scatterplots show mean and standard deviation; ns, *, **, **** denote not significant, *P* < 0.05, *P* < 0.001, and *P* < 0.0001; scale bars represent 20 μm. All data used to plot graphs may be found in Supporting information file S1 Data. GFP, green fluorescent protein; UAS, upstream activating sequence.

In order to confirm that excessive levels of uncleared apoptotic cells in *simu* mutants are responsible for the reduction in cell motility and defects in inflammatory responses to damaged tissue, the ability of cells to undergo developmentally programmed apoptosis was removed from this mutant background using the *Df(3L)H99* deficiency [24]. Comparing apoptosis-null *simu* mutants (i.e., *simu*^*2*^;*Df(3L)H99* double mutants) with *simu* mutants (*simu*^*2*^ single mutants) revealed a rescue of macrophage migration speed (Fig 6a and 6b), whereas there was no difference in migration speed between macrophages in control and apoptosis-null embryos (Fig 6a and 6b; S8 Movie). As expected no vacuoles were seen in macrophages within apoptosis-null embryos, confirming absence of apoptosis and efferocytosis (Fig 6a). Thus, migration defects in *simu* mutants are specifically due to apoptosis and not a subtle morphogenetic or macrophage specification defect.

Next, we sought to test if removal of apoptosis from a *simu* mutant background might rescue inflammatory responses to sites of tissue damage. This represented a more difficult challenge since apoptosis-null embryos themselves exhibit a wound response defect [12], albeit presenting a less severe phenotype than *simu* mutants themselves (Fig 6c and 6d). Quantifying the wound response at 60 minutes post wounding showed no difference between *simu* and apoptosis-null *simu* mutant embryos (Fig 6c and 6d). However, the percentage of macrophages responding to wounds showed a substantial rescue of this initial response to wounds in apoptosis-null *simu* mutant embryos compared to *simu* mutants (Fig 6e; S9 Movie). This rescue of macrophage responses also highlights the fact that wound signals remain intact in *simu* mutants (as per S2f–S2i Fig). Thus, initial inflammatory responses to injury are significantly rescued in *simu* mutants in the absence of apoptosis, suggesting that *simu* is unlikely to play a critical role in the detection or migration of macrophages to wounds. Instead, these data suggest that excessive apoptosis subverts normal macrophage responses to wounds via a distinct mechanism.

**Fig 7:**
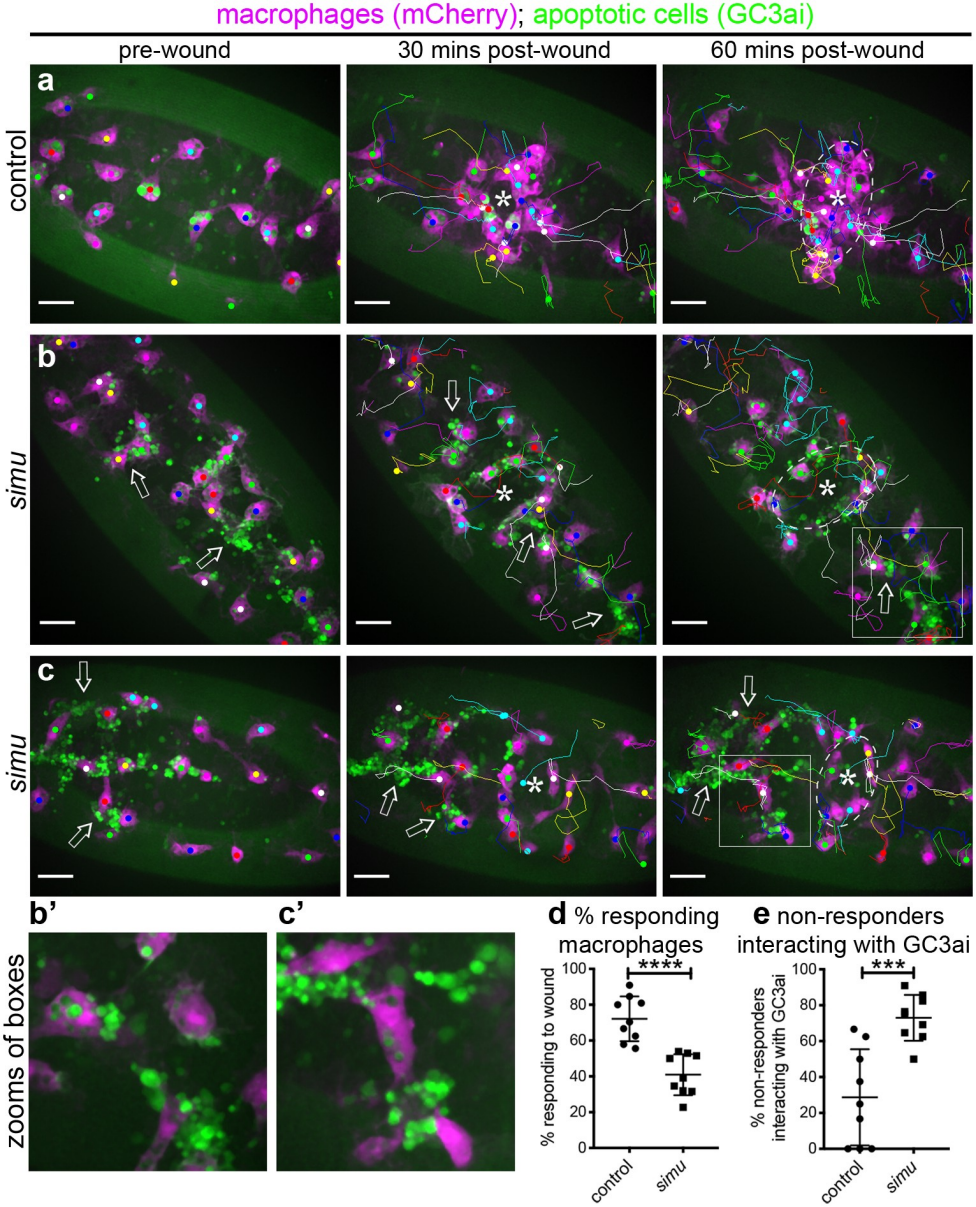
Macrophages that fail to respond to wounds in *simu* mutants interact with uncleared apoptotic cells. (a–c) Ventral projections of the midline region and associated tracks of macrophages (mCherry, purple) migrating to wounds in control (a) and *simu* mutant embryos (b–c) in the presence of the caspase reporter GC3ai (green) to label apoptotic cells/fragments at the indicated times. Asterisks show centre of wound and dotted white lines show wound edges at 60 minutes; arrows indicate examples of regions in which interactions between GC3ai punctae and macrophages occur, disrupting wound responses. (b’–c’) Show zooms and single-channel images of the boxed regions in (b–c). (d–e) Percentage of cells responding to wounding (d) and proportions of nonresponding cells that interact with GC3ai punctae (e) in control and *simu* mutant embryos (*n* = 9 for each genotype, *P* < 0.0001 [d] and *P* = 0.0007 [e], via Mann–Whitney test). All genotypes are *w;;da-GAL4*,*UAS-GC3ai/srp-3x-mCherry* (control) or *w;simu*^*2*^;*da-GAL4*,*UAS-GC3ai/srp-3x-mCherry* (*simu*). N.b., images have not been rotated in order to provide a larger field of view to visualise a greater number of macrophage–apoptotic cell interactions; scale bars represent 20 μm; lines and error bars on (d–e) show mean and standard deviation; *** and **** denote *P* < 0.001 and *P* < 0.0001. All data used to plot graphs may be found in Supporting information file S1 Data. UAS, upstream activating sequence.

Simultaneously imaging macrophage wound responses and apoptotic cell death (via GC3ai) showed that, as shown previously, control responses were very robust and few apoptotic cells were visible (Fig 7a; S10 Movie). In contrast, a varying amount of uncleared apopototic cells were visible in *simu* mutants, with localisation also varying significantly (Fig 7a–7c and S4a and S4b Fig). Clear associations between macrophages that fail to respond to wounds and uncleared apoptotic cells could be seen (Fig 7b and 7c; S11 Movie), suggesting that uncleared apoptotic cells can distract macrophages from their normal migration to wounds. Indeed, the majority of nonresponding macrophages in *simu* mutants interact with uncleared apoptotic cells (Fig 7d and 7e). Consistently, many macrophages undergoing inflammatory migration to wounds in *simu* mutants do so with comparable speeds and directionalities to wild-type cells (S4c–S4g Fig). This suggests that these cells can chemotax efficiently but that some are subverted from their normal behaviours by pathological levels of apoptosis. Thus, while some macrophages fail to respond at all in *simu* mutants, others take a more circuitous route via uncleared apopotic cells to reach wounds.

### Simu mediates phagocytosis of debris at wounds and is required to retain macrophages at sites of tissue injury

**Fig 8:**
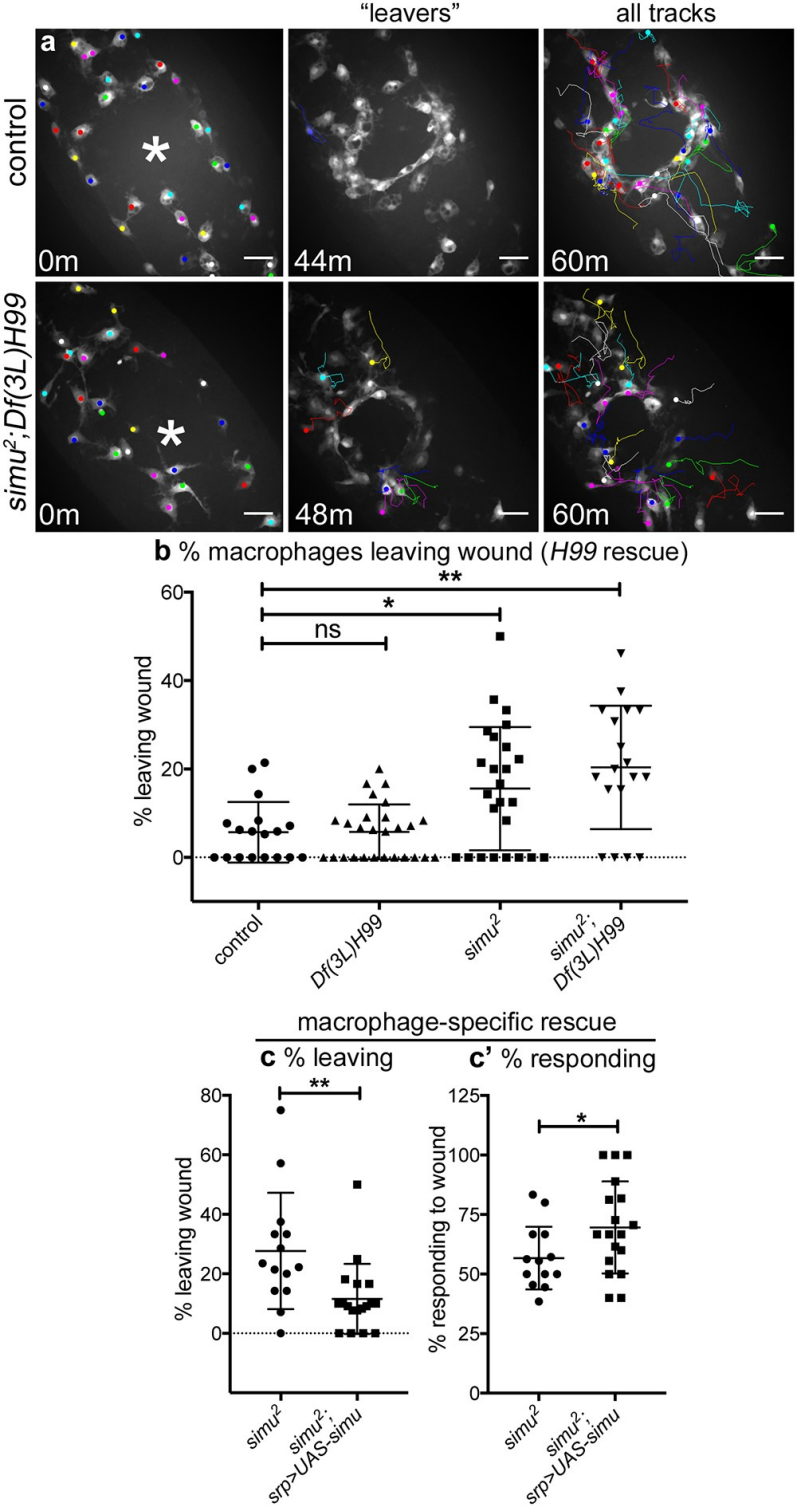
*Simu* is required to prevent precocious exit of macrophages from sites of inflammation. (a) GFP-labelled macrophages and their associated tracks at 0, 44, or 48 and 60 minutes post wounding in controls and *simu* mutants lacking apoptosis (*simu*^*2*^;*Df(3L)H99*). Left panels show macrophages present in field of view at 0 minutes; asterisk marks centre of wound. Central panels show tracks of macrophages that migrate to and then leave the wound (‘leavers’). Right panels show tracks of cells present at 0 minutes that remain in the field of view until 60-minutes post wounding. (b–c) Scatterplots showing percentage of macrophages that migrate away from wounds over the course of 60-minute movies of inflammatory responses in controls, apoptosis-null embryos (*Df(3L)H99*), *simu* mutants (*simu*^*2*^), and *simu* mutants lacking apoptosis (*simu*^*2*^;*Df(3L)H99*) (b) and upon macrophage-specific re-expression of wild-type *simu* in a *simu* mutant background (c); *P* values compared to control in (b) are *P* = 0.999, 0.047, and 0.031, respectively from left to right (Kruskal–Wallis test with Dunn’s multiple comparisons post-test; *n* = 18, 27, 25, 18); for (c) *P* = 0.004 (Mann–Whitney test, *n* = 14 and 18). (c’) Scatterplot of percentages of cells responding to wounds in *simu* mutants and *simu* mutants with re-expression of wild-type *simu* in macrophages (*n* = 14 and 18, *P* = 0.045, Mann–Whitney test). Genotypes are as follows: (a–b) control (*w;;crq-GAL4*,*UAS-GFP*), *Df(3L)H99* (*w;;Df(3L)H99*,*crq-GAL4*,*UAS-GFP*), *simu*^*2*^ (*w;simu*^*2*^;*crq-GAL4*,*UAS-GFP*), and *simu*^*2*^;*Df(3L)H99* (*w;simu*^*2*^;*Df(3L)H99*,*crq-GAL4*,*UAS-GFP*); (c) *simu*^*2*^ (*w;simu*^*2*^,*srp-GAL4*,*UAS-red stinger/simu*^*2*^;*crq-GAL4*,*UAS-GFP/+*) and *simu*^*2*^;*UAS-simu* (*w;simu*^*2*^,*srp-GAL4*,*UAS-red stinger/simu*^*2*^;*crq-GAL4*,*UAS-GFP/UAS-simu*). Lines and error bars show mean and standard deviation in scatterplots; scale bars represent 20 μm (a). All data used to plot graphs may be found in Supporting information file S1 Data. GFP, green fluorescent protein; UAS, upstream activating sequence.

An abnormal number of macrophages were present at wounds 60 minutes after wounding in *simu* mutants devoid of apoptosis despite a normal initial migratory response to wounds. Why then is there a defect in wound responses at later timepoints? Vertebrate innate immune cells are removed from sites of inflammation by either apoptosis [3,40] or reverse migration [41–44], but there is no evidence to suggest that *Drosophila* macrophages die at wounds. Consequently, we investigated whether precocious exit from sites of damage explained the incomplete rescue of wound responses in *simu* mutants in the absence of apoptosis. Macrophages rarely left control wounds in the 60-minute post wounding; by contrast, significantly more macrophages failed to remain at wounds in both *simu* and apoptosis-null *simu* mutant embryos compared to controls (Fig 8a and 8b; S12 Movie). Unlike defects in initial migratory responses to wounding (Fig 6e), this phenotype was maintained in the absence of apoptosis (Fig 8b), suggesting that uncleared apoptotic cells were not drawing macrophages away from wounds but rather *simu* regulates retention of macrophages at wounds. Macrophage-specific re-expression of *simu* rescued the percentage of macrophages migrating to wounds and also decreased exit from wounds in *simu* mutants (Fig 8c and 8c’). This indicates that *simu* functions autonomously within macrophages and is necessary and sufficient to maintain these cells at sites of tissue damage. To our knowledge, this represents the first example of a gene controlling retention of macrophages at wounds in *Drosophila* embryos.

Surprisingly, loss of *simu* does not affect survival and hatching of wounded embryos (S5a and S5b Fig) or prevent activation of signalling pathways associated with inflammation (e.g., c-Jun N-terminal kinase [JNK] signalling [45]; S5c and S5d Fig). However, the reduction in recruitment and early exit of macrophages from wounds results in a more rapid termination of the macrophage inflammatory response (S5e Fig), which is surprisingly associated with enhanced rates of wound closure (S5f Fig). Therefore, it is possible that recruitment of innate immune cells can hamper repair processes within *Drosophila* embryos.

**Fig 9:**
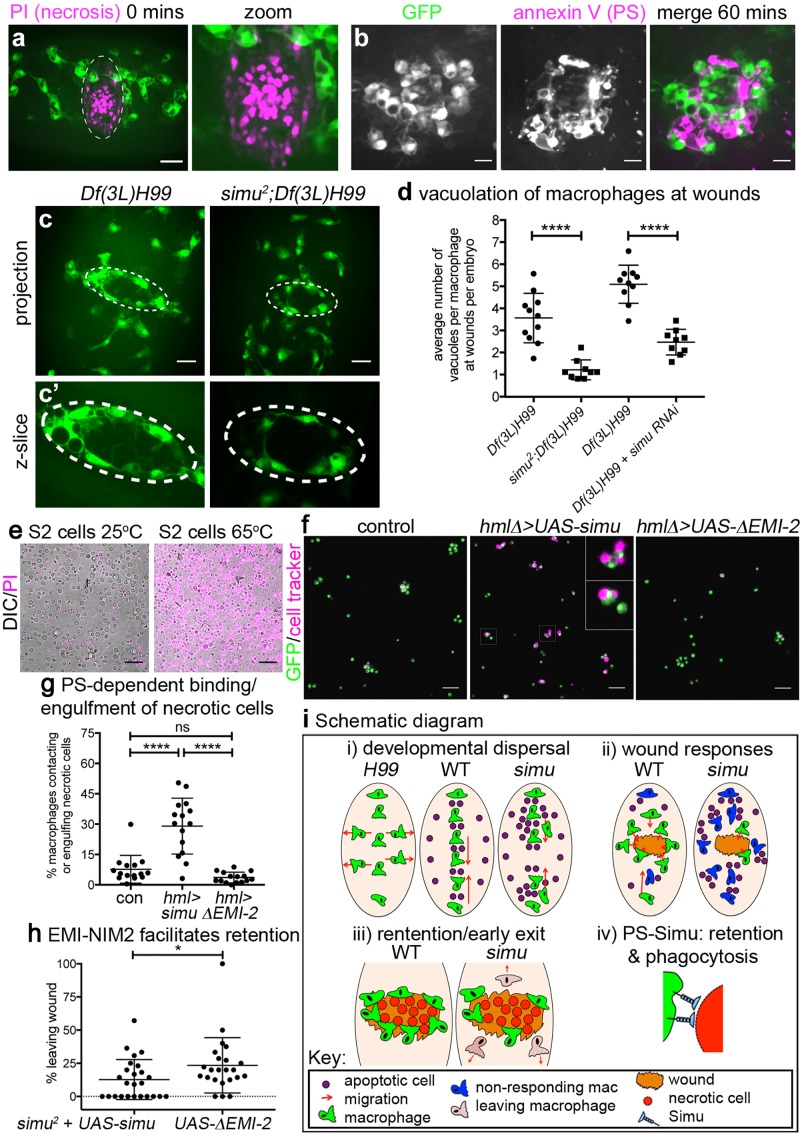
*Simu* recognises and facilitates removal of necrotic debris at wounds in a PS-dependent manner, preventing early exit of macrophages from sites of inflammation. (a) PI staining to show necrotic cells immediately after wounding in a stage 15 control embryo (GFP-labelled macrophages, green; PI, purple). Dotted lines in left-hand panel show wound edge; right-hand panel shows zoom of wound. (b) Panels show GFP-labelled macrophages (green in merged image), PS staining (via annexin V, purple in merged image) and merged image at a wound site at 60-minutes post wounding of a stage 15 control embryo. (c) Maximum projections of GFP-labelled macrophages in apoptosis-null embryos (*Df(3L)H99*) and *simu* mutants that lack apoptosis (*simu*^*2*^;*Df(3L)H99*) at 60-minutes post wounding; note absence of vacuoles in macrophages away from the wound (indicated via dotted line). (c’) Shows zooms of wound regions taken from single z-slices from image stacks used to make projections in (c). (d) Scatterplot comparing numbers of vacuoles present in macrophages at wounds in apoptosis-null embryos (*Df(3L)H99*) with apoptosis-null embryos that lack *simu* (*simu*^*2*^;*Df(3L)H99*) (*n* = 11 and 9, respectively; *P* < 0.0001, Mann–Whitney test), and apoptosis null embryos with (*Df(3L)H99 + simu RNAi*) or without (*Df(3L)H99*) macrophage-specific RNAi-mediated knockdown of *simu* (*n* = 10 and 9, respectively; *P* < 0.0001, Mann–Whitney test). (e) Heat treatment of S2 cells causes all cells to undergo necrosis and become labelled with propidium iodide (purple). (f) Images showing that expression of wild-type *simu* but not a truncated form of *simu* that cannot bind PS (ΔEMI-2) increases recognition of necrotic S2 cells by larval macrophages compared to controls. *hmlΔ-GAL4* used drive larval macrophage-specific expression of *UAS-GFP* (green) and *UAS-simu* constructs; S2 cells labelled using cell tracker (purple) prior to induction of necrosis; inset shows zoomed examples of macrophages binding and engulfing necrotic S2 cells (indicated by boxes in main panel). (g) Scatterplot showing quantification of binding and/or engulfment of necrotic cells by larval macrophages (*n* = 15 per genotype, *P* < 0.0001 [control versus *UAS-simu*], *P* = 0.433 [control versus *UAS-ΔEMI-2*] and *P* < 0.0001 [*UAS-simu* versus *UAS-simuΔEMI-2*], one-way ANOVA). (h) Scatterplot showing that macrophage-specific expression of *simu ΔEMI-2* leads to enhanced exit of macrophages from wound sites compared to wild-type *simu* in a *simu* mutant background (*w;simu*^*2*^,*srp-GAL4*,*UAS-red stinger*,*Ecad-mCherry/simu*^*2*^;*crq-GAL4*,*UAS-GFP/UAS-simu or UAS-simu ΔEMI-2*; *n* = 26 and 21, respectively, *P* = 0.039, Mann–Whitney test). (i) Schematic summarising roles of apoptotic cells and Simu in macrophage dispersal, migration, retention at wounds, and phagocytosis. Lines and error bars on graphs show mean and standard deviation; *, ****, and ns represent *P* < 0.05, *P* < 0.0001, and not significant; scale bars represent 20 μm (a, c) and 10 μm (b) and 50 μm (e–f). All data used to plot graphs may be found in Supporting information file S1 Data. GFP, green fluorescent protein; PI, propidium iodide; PS, phosphatidylserine; RNAi, RNA interference; UAS, upstream activating sequence.

To understand how Simu mediates retention of macrophages at sites of injury, we investigated the wound environment in more detail: laser ablation results in large amounts of necrotic death at wounds, as revealed by instantaneous entry of propidium iodide (PI) into ruptured cells at these sites of damage (Fig 9a; S6a–S6c Fig). This loss of membrane integrity leads to externalisation of PS (Fig 9b; S6d Fig), a known ligand of Simu [22]. PS staining accumulates at sites of damage, particularly at the wound margin, and indicates the persistence of large amounts of debris, even at 60 minutes post wounding (Fig 9b, S6d Fig and S13 Movie). We hypothesised that macrophages are retained at wounds through either inhibition of cell migration signals downstream of Simu or via physical interactions between PS and Simu at these sites of damage. Consistent with interaction of Simu and PS-positive necrotic debris at wounds, macrophages phagocytosed large amounts of debris at these sites in apoptosis-null embryos but not in those embryos that lacked both *simu* function and apoptotic cell death or when *simu* was removed specifically from macrophages via RNA interference (RNAi) (Fig 9c and 9d). Thus, these data reveal another novel function for Simu in that it is required in macrophages for phagocytosis of nonapoptotic debris at wounds, suggesting a more general role in clearance of damaged cells in vivo rather than acting specifically in efferocytosis.

Finally, we hypothesised that Simu–PS interactions mediate engulfment of necrotic cells and retention of macrophages at wounds. Expression of full-length *simu* but not a version of *simu* lacking the domains required to bind PS (EMI-NIM2) [22] was sufficient to stimulate binding and engulfment of necrotic S2 cells by larval macrophages in vitro (Fig 9e–9g). Similarly, macrophage-specific expression of *simuΔEMI-NIM2-GFP* failed to prevent early exit of macrophages from wounds in *simu* mutants (Fig 9h). These data therefore suggest that Simu–PS interactions directly mediate binding and engulfment of necrotic cells and contribute to retention of macrophages at sites of injury.

## Discussion

Here, we show that developmentally programmed apoptosis contributes to dispersal of macrophages in fly embryos, while excessive amounts of apoptosis disrupts macrophage dispersal, migration, and inflammatory responses to wounds (see schematic diagram in Fig 9i). Acute induction of apoptosis phenocopied defects in inflammatory responses, suggesting that apoptotic cells drive these changes in macrophage behaviour and can do so independently of phagocytosis. Importantly, removal of apoptosis from a *simu* mutant background rescued migration and the initial inflammatory recruitment to wounds. These investigations reveal a novel role for Simu in retention of macrophages at wounds, which depends on the PS-binding regions of Simu, and suggests that this receptor functions more generally to facilitate clearance of both apoptotic and necrotic cells.

We found that macrophage dispersal was impaired in the absence of apoptosis with precocious migration from the ventral midline leading to reduced numbers localised on the developing CNS. Macrophage specification and migratory ability appeared otherwise normal in the absence of apoptosis since cells were able to migrate at normal speeds. Dying cells may therefore provide instructive cues to aid dispersal. Indeed, apoptotic cells are known to play a role in recruitment of tissue-resident macrophages in other systems [46,47]. Apoptotic midline cells [48] may act to retain macrophages on the midline in wild-type embryos, potentially explaining why macrophages migrated prematurely from the midline in the absence of apoptosis. Alternatively, factors released by ‘undead’ cells (cells induced to die but in which execution of apoptosis is blocked) may interfere with macrophage dispersal in the absence of apoptosis. Indeed, ‘undead’ cells are known to release signalling molecules such as transforming growth factor β (TGFβ) family proteins and H_2_O_2_ [49,50], known regulators of macrophage behavior in many disease states that are conserved across different models [14,51]. In contrast, interactions with uncleared apoptotic cells appear to slow migration, also leading to aberrant dispersal. Morphological defects are unlikely to contribute since *simu* mutants appear grossly normal and are viable [23].

We found that apoptotic cell death was necessary for impairment of migration and wound responses in *simu* mutants, in which uncleared apoptotic cells surround macrophages. Furthermore, we could reproduce wound recruitment defects by acute induction of apoptosis with macrophages failing to migrate to wounds. Phagocytosis of dying cells was not required for this effect; consequently, we favour a model whereby ‘find-me’ signals released from uncleared apoptotic cells act to subvert normal macrophage responses. Find-me cues are signals released by dying cells to recruit phagocytes to facilitate clearance [52], though none have been identified to date in *Drosophila*. Consistent with this hypothesis, live imaging of apoptosis and macrophage migration during wound responses indicate that the majority of macrophages failing to respond to wounds in *simu* mutants interact with clusters of uncleared apoptotic cells. The release of find-me cues or other stress signals produced in response to uncleared apoptotic cells may confuse chemotactic responses of macrophages by operating as competing sources of chemoattractant or attenuating signalling events required for other responses, as seen in signal priorisation by neutrophils during their inflammatory recruitment [53].

We found that, on average, macrophages migrated more slowly in *simu* mutants; however, many cells can move at comparable speeds to their wild-type counterparts, suggesting that the general migration machinery is not greatly perturbed. This slowed migration seems unlikely to account for impaired wound responses in *simu* mutants since macrophages lacking β-integrin move more slowly but still ultimately reach wounds in normal numbers [54]. In addition to the large increase in uncleared apoptotic cells, there was also a small increase in the phagocytic index of macrophages, although this only equated to approximately one extra puncta per cell. Others have reported that vacuolation of innate immune cells is associated with defective migration [55,56], while overloading macrophages with engulfed cargoes can lead to phagosomal maturation defects [57]. Furthermore, other professional phagocytes such as *Dictyostelium* amoebae and dendritic cells pause migration when internalising cargo, albeit through macropinocytic mechanisms [58,59]. However, we did not observe obvious repeated failures of phagocytic events by *simu* mutant macrophages, suggesting that attempts to phagocytose uncleared apoptotic cells were not stalling migration. Macrophages in *simu* mutants are significantly less vacuolated than suppressor of cyclic AMP receptor/WASP-family verprolin homologous protein (*SCAR/WAVE*) complex mutant *Drosophila* macrophages and lysosomal storage mutant macrophages in zebrafish larvae [55,56]—whether subtle changes in the numbers of phagosomes seen in this study can account for such potent disruption of macrophage behaviour seems unlikely from the point of view of evolution of a functional innate immune system, but the threshold at which phagosomal maturation becomes pathological has yet to be directly addressed. An alternative explanation would be promotion of an anti-inflammatory state of programming of macrophages by apoptotic cell clearance [60]. Currently, there is no strong evidence for such macrophage programming in *Drosophila*, though some progress has been made by Ando and colleagues [61,62]. Nonetheless, clearance of apoptotic cells is associated with increased expression of phagocytic receptors in a number of phagocytic cell types, including macrophages [12,63–65]. *Drosophila* embryonic macrophages also undergo remarkable shifts in behaviour over the fly life cycle, controlled in part by lipid hormone signalling [66,67]. However, the anti-inflammatory phenotype we observed on acute induction of apoptosis would necessitate rapid reprogramming of macrophages, which seems unlikely for lipid hormone signalling. This therefore points towards a more instantaneous mechanism, such as the release of find-me cues.

Laser wounding of the epithelium led to accumulation of necrotic debris at sites of injury, including an accumulation and persistence of a marker used by phagocytes to clear apoptotic cells (PS). Simu binds PS via three N-terminal domains [22] and is necessary for normal phagocytosis of necrotic debris at wounds. This work suggests that Simu is a receptor for both apoptotic and necrotic cells and should be reclassified as a more general scavenger receptor, as for the *Drosophila* receptors Draper and Croquemort [63,68,69]. Removal of uncleared apoptotic cells from a *simu* mutant background rescued migration and improved responses to wounds. The early departure of macrophages from such wounds resembles reverse migration of neutrophils [41,44] and exit of macrophages, which can leave sites of inflammation via lymphatic vessels in vertebrates [43,70]. This failure of retention contributes to the impaired macrophage inflammatory responses seen in the absence of Simu and undermines the rescue of wound responses seen in the absence of apoptosis (*simu;Df(3L)H99* double mutants). The PS-binding domain of Simu is required to maintain macrophages at wound sites, therefore Simu may directly mediate retention of macrophages at wounds via binding to PS and/or other ligands. Alternatively, Simu may act more indirectly, possibly instructing macrophages to remain at wounds by facilitating signalling through other coreceptors. The fact that initial responses to wounds were rescued in the absence of apoptosis implies that Simu is not interpreting chemotactic cues released from wounds, while precocious exit of macrophages from wounds in the absence of both Simu and apoptotic cell death (*simu*^*2*^;*Df(3L)H99* mutants) suggests these phagocytes are not simply being distracted away by apoptotic cells.

In summary, we show that the presence of apoptotic cells significantly alters macrophage behaviour in vivo and reveal new functions for the apoptotic cell receptor Simu. Our finding that pathological levels of apoptosis impair multiple macrophage functions has significant implications for a wide range of human conditions in which changes in apoptotic cell death and efferocytosis occur and are accompanied by aberrant or subverted macrophage behaviour that drives or exacerbates disease progression, such as cancer, atherosclerosis, neurodegenerative disorders, and other chronic inflammatory conditions.

## Materials and methods

### Fly genetics and husbandry

*Drosophila melanogaster* fruit flies were reared on standard cornmeal/agar/molasses media at 18 °C or 25 °C. Embryos were harvested from laying cages with apple juice agar plates left overnight at 22 °C. *Srp-GAL4* and/or *crq-GAL4* were used to label embryonic macrophages with GFP, red stinger and/or CD4-tdTomato, or to drive expression of other transgenes; *srp-3x-mCherry* [71] was used to label macrophages in a GAL4-independent manner. *hmlΔ-GAL4* [72] was used to drive expression of UAS transgenes in larval macrophages. The following alleles, transgenes, and deficiencies were used in this study: *srp-GAL4* [7], *crq-GAL4* [36], *da-GAL4* [73], *hmlΔ-GAL4* [72], *srp-3x-mCherry* [71], *Ecad-mCherry* [74], *UAS-GFP*, *UAS-nls-GFP*, *UAS-red stinger* [75], *UAS-CD4-tdTomato* [76], *UAS-2xFYVE-GFP* [28], *UAS-apoliner* [39], *UAS-GCamP6M* [37], *UAS-GC3ai* [34], *UAS-simu* [23], *UAS-simuΔEMI-NIM2-GFP* [22], *UAS-simu RNAi* (TRiP line HMJ23355) [77], *simu*^*2*^ [23], *Df(3L)H99* [24], *Df(2L)BSC253* [78], *CyO hs-hid* [38]. Selection against the fluorescent balancers *CTG*, *CyO dfd*, *TTG*, or *TM6b dfd* was used to discriminate homozygous mutant embryos for recessive lethal alleles [79,80]. See S1 Table for a full list of genotypes used in this study and their sources.

### Imaging and wounding of *Drosophila* embryos

Embryos were washed off apple juice agar plates, dechorionated in bleach, and then washed in distilled water. Live and fixed embryos were mounted on slides in voltalef oil (VWR) or DABCO (Sigma), respectively, as per Evans and colleagues, 2010 [9]. Immunostained embryos were imaged on a Nikon A1 confocal system using a 40X objective lens (CFI Super Plan Fluor ELWD 40x, NA 0.6), which was used for the migration movies in Fig 4. Aside from S2 Movie (Zeiss Lightsheet system; see S1 Methods), all other timelapse imaging and wounding was performed on a Perkin Elmer UltraView Spinning Disk system using a 40X objective lens (UplanSApo 40x oil, NA 1.3). Lower magnification images of embryos were taken using a MZ205 FA fluorescent dissection microscope with a PLANAPO 2X objective lens (Leica) or a 20x objective (UplanSApo 20x, NA 0.8) on the Perkin Elmer UltraView Spinning Disk system.

### Fixation and immunostaining of *Drosophila* embryos

Embryos from overnight plates were fixed and stained as per Evans and colleagues, 2010 [9]. Antibodies were diluted 1:1 in glycerol for storage at −20 °C and these glycerol stocks were diluted as indicated in phosphate-buffered saline (PBS; Oxoid) containing 1% BSA (Sigma) and 0.1% Triton-X100 (Sigma). Rabbit anti-cDCP-1 (1:500; 9578S –Cell Signaling Technologies), 22C10 mouse anti-Futch, (supernatant used at 1:100), 8D12 mouse anti-Repo, (concentrate used at 1:500 –Developmental Studies Hybridoma Bank) were used as primary antibodies. Rabbit anti-GFP (1:500; ab290 –Abcam) or mouse anti-GFP (1:100; ab1218 –Abcam) were used to stain GFP-expressing macrophages for co-immunostaining with mouse and rabbit primary antibodies, respectively. Fluorescently-conjugated goat anti-mouse or goat anti-rabbit secondary antibodies (Alexa Fluor 568, Alexa Fluor 488 –Thermo Fisher, or FITC–Jackson Immunoresearch) were used to detect primary antibodies at 1:200.

### Macrophage migration assays

Embryos with fluorescently labelled macrophages were mounted ventral-side up and left to acclimatise on slides for 30 minutes before imaging for all migration assays. Macrophage behaviour was analysed from stage 12 (developmental dispersal), stage 13 (lateral migration), or stage 15 (wounding and wandering/random migration). Z-stacks correspond to the region between the epithelium and CNS. Maximum projections of despeckled z-stacks were assembled and tracked using the manual tracking plugin in Fiji [81,82]. For random migration analysis, only those macrophages lying between the edges of the VNC at the start of the movie were tracked, with cells imaged every 2 minutes for 1 hour (with the exception of data in Fig 4, which was collected every 1 minute for a 30-minute period). Individual cell speeds and directionalities were calculated using the Chemotaxis plugin (Ibidi) in Fiji.

For the wounding assay, prewound z-stacks were taken prior to wounding of stage 15 embryos on their epithelial surface using a nitrogen-pumped Micropoint ablation laser (Andor), as per Evans et al., 2015 [16]. Z-stacks of wounded embryos were collected every 2 minutes for 1 hour (or every 20 minutes for 3 hours for S5e and S5f Fig). At the end of the movie, an additional z-stack was taken incorporating a brightfield image with which to measure the wound size (60-minute movies). Movies were assembled as per wandering migration movies. The number of macrophages at wounds was quantified from a 15 μm deep z-stack, with the first z-slice used in this analysis corresponding to the one containing the most superficial macrophage adjacent to but not at the wound. Wound area was measured from the brightfield (or Ecad-mCherry channel; S5e and S5f Fig). Macrophage responses to wounds were calculated as follows: number of macrophages within or touching the wound area divided by wound area in μm^2^. This was normalised according to control responses.

To calculate the initial inflammatory response to wounds, the percentage of responding macrophages was calculated: this is the percentage of macrophages present at in the field of view at 0 minutes post wounding that migrated to the wound; macrophages already present at the wound site at this timepoint were not counted as responding nor included in the total numbers present in the field of view. Macrophages that left the wound were not counted a second time if they returned and were defined as responding if they moved towards and touched the wound edge.

To assess exit of macrophages from wound sites, the percentage of macrophages that left the wound site was counted. This is the proportion of macrophages (either that were present at the wound site at *t* = 0 minutes or that reached the wound at any point during the wound movie) that left the wound during the course of a 60-minute timelapse movie; if any part of a macrophage retained contact with the wound edge, it was not scored as having left.

### Introduction of exogenous apoptosis via heat-shock

*Hs-hid* or control embryos from overnight plates were washed off into embryo baskets (70μm cell strainers, Fisher) using distilled water. Baskets containing embryos to be heat-shocked were placed into a large weighing boat containing 75ml of prewarmed distilled water in a circulating water bath set to 39 °C for the indicated time. Non heat-shocked controls were incubated in distilled water at room temperature. Following heat shock, embryos were dechorionated and then stage 15 embryos selected and mounted for live imaging 30 minutes before they were due to be wounded, such that they could acclimatise on slides. For instance, if embryos were wounded at 60 minutes post heat shock, slides were set up to be ready for 30 minutes post heat shock. Embryos were then wounded and imaged as described above to visualise macrophage behaviour or caspase activity via the apoliner reporter [39]. Alternatively, embryos were fixed at various timepoints post heat shock to reveal caspase activity via cDCP-1 immunostaining.

### Injection of propidium iodide and annexin V

PI and annexin V are well-characterised reagents used to detect membrane permeabilisation/necrosis and exposure of PS/apoptosis, respectively [83,84]. Embryos were mounted ventral-side up on double-sided sticky tape (Scotch), dehydrated for 7–10 minutes in an air-tight box containing sachets of silica gel (Sigma), then covered in voltalef oil before microinjection. Borosilicate glass needles (World Precision Instruments; capillaries were 1-mm and 0.75-mm inner and outer diameter capillaries, respectively) for microinjection were made using a P1000 needle puller (Sutter). Embryos were microinjected anteriorly into the head region with 1 mg/ml PI (Sigma) dissolved in PBS or into the vitelline space with undiluted Alexa Fluor 568-labelled Annexin V (Invitrogen). After injection, a coverslip (thickness 1, VWR) was applied, supported by two coverslip bridges (thickness 1, VWR) placed on either side of the embryos, and fixed in place with nail varnish. Embryos were then imaged immediately, wounded, and then reimaged to detect membrane permeabilisation and/or externalisation of PS. As a positive control for necrotic cell death, embryos were injected with PI, coverslips attached, and then heated at 60 °C in a humidified box for 15 minutes.

### Challenge of larval macrophages with necrotic cells

*Drosophila* S2 cells were grown in Schneider’s medium (Sigma) supplemented with 10% heat-inactivated FBS (Thermo Fisher) and 1X Pen/Strep (Thermo Fisher). S2 cells were labelled via incubation for 30 minutes with 5 μM cell tracker red (Thermo Fisher). Cell tracker red-labelled S2 cells were then washed off tissue culture flasks and numbers counted using a haemocytometer. Residual cell tracker was removed by centrifugation of resuspended cells at 1,350 rpm for 5 minutes at 4 °C; cells were resuspended in fresh S2 media at a concentration of 4.5 x 10^5^ cells per ml and heated in 0.5 ml aliquots at 65 °C for 10 minutes to induce necrosis (adapted from Kimura and colleagues, 2014 [85]). Necrosis was confirmed by heat treating of nonstained cells in the presence of 0.5 μg/ml PI (Biolegend). Necrotic cell suspensions were cooled to room temperature in a water bath and then 75 μL of the necrotic cell suspension (3.4 x 10^4^ cells; an estimated ratio of 5:1 necrotic cells per larval macrophage that is based upon average densities of cells dissected from *w*^*1118*^ larvae) was added per well to challenge adhered larval macrophages. Macrophages were obtained by dissecting a single L3 wandering larvae of the indicated genotype in 75 μL S2 media on ice, before transferring macrophage suspensions to a well in a Porvair 96-well plate to adhere for 45 minutes ahead of challenge with necrotic cells. Necrotic-larval macrophage cocultures were left for 1 hour to allow binding/engufment before removal of media and fixation using 4% formaldehyde in PBS for 15 minutes. Fixed cells were imaged using a Nikon Ti Eclipse system with a CFI Plan Apochromat λ 20X (NA 0.75) objective lens. The proportion of GFP-labelled larval macrophages binding/engulfing necrotic, cell tracker red-labelled S2 cells was then scored from blinded images and fields of view averaged per well (5 fields of view per well, 1 larva per well).

### Quantification of phagocytosis and failed apoptotic cell clearance

Anti-cDCP-1 and anti-GFP immunostained embryos were imaged on a Nikon A1 system using the 40X objective lens with a spacing of 0.25 μm between z-slices. Channels were merged to enable discrimination of cDCP-1 punctae within/outside macrophages. A portion of the z-stack representing a 10 μm deep volume corresponding to the area occupied by macrophages between the epithelium and VNC was used to count numbers of cDCP-1 punctae per macrophage to quantify phagocytic index. Only macrophages completely in view in the 10 μm substack were included in the analysis and these values were averaged to give an overall value per embryo; at least 10 macrophages were analysed per embryo. Untouched apoptotic cells were quantified by counting the number of red cDCP-1 punctae that are located within the 10 μm substack, but that were not in contact with GFP-labelled macrophages. This method was selected to enable comparison with the quantification of Kurant and colleagues, 2008 [23] and also since it was technically challenging to discriminate whether cDCP-1 punctae in close contact with macrophages were inside or outside of macrophages in *simu* mutants owing to the extreme buildup of uncleared apoptotic cells in this background.

Number of vacuoles per macrophage (averaged per embryo) was used as an indirect read-out of phagocytosis. *Drosophila* macrophages exclude cytoplasmic GFP from phagosomes and these can be shown to correspond to apoptotic cells by their absence from macrophages in apoptosis-null *Df(3L)H99* embryos. Vacuoles were counted using z-stacks of GFP-labelled macrophages taken from live imaging experiments. Vacuoles were assessed in the z-slice in which each macrophage exhibited its maximal cross-sectional area. Therefore, this analysis should not be considered as the absolute numbers of corpses per cell but a relative read-out thereof.

Phagocytosis was also quantified from stills or movies of macrophages expressing either 2xFYVE-GFP or CD4-tdTomato. Z-stacks were taken every 15 seconds for 20 minutes. Phagocytic events were scored when new phagocytic events occurred via formation of phagocytic cups (CD4-tdTomato) or the numbers of 2xFYVE-GFP punctae larger than 2 μm with an obvious lumen present in macrophages, as a read-out of recently formed phagosomes. 2xFYVE-GFP punctae of this size are absent from macrophages in *Df(3L)H99* mutants.

### Analysis of developmental dispersal and macrophage numbers

Developmental dispersal of macrophages was analysed by scoring the presence of GFP-labelled macrophages between the epithelium and VNC in each segment at stage 13/14 following fixation and immunostaining for GFP. Embryos with macrophages in all segments were scored as exhibiting 100% progression along the midline; an embryo that lacked macrophages in segments 6–9 would have been scored as 66% progression.

As a read-out of total numbers of macrophages per embryo, lateral views of embryos containing red stinger-labelled macrophages were stitched together, thresholded and processed using the watershed tool in Fiji. The analyse particles tool was then used to count cells. Lateral views correspond to a depth of 50 μm from where the most superficial macrophages come into focus. At other stages of development, numbers of GFP-labelled macrophages per field of view between the epithelium and VNC were scored.

### Image processing, quantification, and statistical analyses

Image processing was carried out in Fiji/ImageJ, with images despeckled before maximum projections were assembled. Brightness and contrast enhancements were applied equally across all data sets being compared with each other. A Python (Python Software Foundation) script provided by the Whitworth Lab (University of Cambridge) was used to blind images ahead of analysis. Figures were assembled in Photoshop (Adobe).

Statistical analyses were performed using Prism (GraphPad); see legends and text for details of statistical tests used. *N* numbers refer to individual *Drosophila* embryos (unless otherwise stated), taken from laying cages containing >50 adult flies; experiments were conducted across at least three imaging sessions with control embryos mounted on the same slides each time. Previous experimental data shows that >6 movies (wandering migration) and 15–20 wound movies are sufficient to detect an effect size of 20% that of control values. Movies that lost focus or wounds that leaked obscuring macrophages at wounds were excluded from analyses. Immunostaining was performed on batches of pooled embryos collected across multiple days, with controls stained in parallel using staining solutions made up as a master mix and split between genotypes.

## Supporting information

S1 Data Data used to plot all graphs and to perform statistical analyses. (XLSX)

S1 Fig Development and macrophage dispersal in *simu* mutants and validation of Caspase-ON reporters. (a) Average percentages of control and *simu* mutant embryos at indicated developmental stages of following ageing for 28 hours at 18 °C (scored from 3 separate lays of 1.5 hours at 25 °C). No significant difference between proportions of embryos at stage 16 or earlier and stage 17 or later for these genotypes (Fisher’s exact test, *P* = 0.0581, *n* = 418 and 144 embryos, respectively). (b) Average speed per GFP-labelled macrophage, per embryo at each timepoint from late stage 12 onwards (when a single line of macrophages is present on the ventral midline) for 90 minutes in controls and *simu* mutant embryos (*n* = 18 and 16, respectively). (b’) Scatterplot of average speed per macrophage per embryo over 90 minutes from late stage 12. (c) Stills taken from a timelapse movie of *srp-3x-mCherry*-labelled macrophages dispersing along the ventral midline in a control embryo ubiquitously expressing a caspase-ON reporter (GC3ai). (c) Shows overall field of view; (c’) zooms showing onset of GC3ai fluorescence, fragmentation of the dying cell and its subsequent engulfment by a macrophage. (d) Numbers of macrophages on the ventral midline at stage 15 of development in controls (*n* = 23), *Df(3L)H99* mutants (*n* = 18), *simu* mutants (*n* = 23) and *simu;Df(3L)H99* double mutants (*n* = 23). Genotypes are *w;;crq-GAL4*,*UAS-GFP* (control) and *w;simu*^*2*^;*crq-GAL4*,*UAS-GFP* (*simu*) (a–b, d), *w;;Df(3L)H99*, *crq-GAL4*,*UAS-GFP* (*Df(3L)H99*) (d), *w;simu*^*2*^;*Df(3L)H99*,*crq-GAL4*,*UAS-GFP* (*simu;Df(3L)H99*) (d) and *w;;da-GAL4*,*UAS-GC3ai/srp-3x-mCherry* (c). Bars and data points show means, error bars show standard deviation (a, b’, d), or standard error of the mean (b); **, ***, and **** denote *P* < 0.01, *P* < 0.001, and *P* < 0.0001 via Mann–Whitney test (b’) or one-way ANOVA (d); scale bar represents 20 μm (c). All data used to plot graphs may be found in Supporting information file S1 Data. GFP, green fluorescent protein; UAS, upstream activating sequence.
(TIF)

S2 Fig *Simu* has a partially cell autonomous function in macrophages and generation of wound signals are normal in the absence of *simu*. (a–b) Ventral images showing macrophage morphology in control (*w;;crq-GAL4*,*UAS-CD4-tdTomato*) and *simu* mutant embryos (*w;simu*^*2*^;*crq-GAL4*,*UAS-CD4-tdTomato*) at stage 15. (c–e) Scatterplots showing rates of phagocytosis (phagocytic events per hour, per macrophage, per embryo, c), macrophage spread area (μm^2^ per macrophage, d) and macrophage circularity (e). Statistical comparisons made via Mann–Whitney tests; *n* = 12 control and 11 *simu*^*2*^ embryos (c) and *n* = 37 control and 18 *simu*^*2*^ macrophages taken from 11 embryos (d–e). *P* = 0.028, 0.179, 0.0009 (c–e). (f–g) Images of cytoplasmic calcium levels (visualised using GCamP6M) in the epithelium prior to (f–g) and immediately after wounding (f’–g’) in control (*w;;da-GAL4*,*UAS-GCamP6M*, f-f’) and *simu* mutant embryos (*w;simu*^*2*^;*da-GAL4*,*UAS-GCamP6M*, g–g’). (h) Line graph showing timecourses of GCamP6M response (GCamP6M MGV of the entire embryonic field of view at each timepoint normalised to the prewound MGV) in control and *simu* mutant embryos (same genotypes as f–g). (i) Scatterplot of the ratio of initial response (F1) and prewound (F0) MGV in control and *simu* mutant embryos (*n* = 11 and 15, respectively; *P* = 0.80, Mann–Whitney test). (j) Ventral images showing wound responses in control (*w;;crq-GAL4*,*UAS-GFP*), *simu/+* heterozygous (*w;simu*^*2*^*/+;crq-GAL4*,*UAS-GFP*), *Df(2L)BSC253/+* heterozygous (*w;Df(2L)BSC253/+;crq-GAL4*,*UAS-GFP*) and *simu/Df(2L)BSC253* trans-heterozygous embryos (*w;simu*^*2*^*/Df(2L)BSC253;crq-GAL4*,*UAS-GFP*) at 60 minutes post wounding. (k) Scatterplot showing wound responses corresponding to genotypes shown in (j). *N* numbers (left–right) are 19, 20, 19, 16; ns, *, **, ***, and **** denote not significant (*P* = 0.10), *P* = 0.043, 0.0079 and *P* < 0.0001, respectively, via one-way ANOVA with Dunn’s multiple comparisons post-test. (l) Ventral images showing wound responses (macrophages per wound area, normalised to control) at 60 minutes post wounding in controls (*w;srp-GAL4*,*UAS-red stinger/+;crq-GAL4*,*UAS-GFP/+*), *simu* mutants (*w;simu*^*2*^,*srp-GAL4*,*UAS-red stinger/simu*^*2*^;*crq-GAL4*,*UAS-GFP/+*), embryos in which *simu* was re-expressed in macrophages (*w;srp-GAL4*,*UAS-red stinger/+;crq-GAL4*,*UAS-GFP/UAS-simu*) and *simu* mutants in which *simu* was re-expressed in macrophages (*w;simu*^*2*^,*srp-GAL4*,*UAS-red stinger/simu*^*2*^;*crq-GAL4*,*UAS-GFP/UAS-simu*). (m) Scatterplot showing wound responses corresponding to genotypes in (l). *N* numbers (left–right) are 14, 15, 16, 18; ns and * denote not significant (*P* = 0.27) and *P* = 0.017, via Mann–Whitney tests. Lines and error bars show mean and standard deviation on scatterplots; scale bars represent 20 μm. All data used to plot graphs may be found in Supporting information file S1 Data. GFP, green fluorescent protein; MGV, mean gray value; UAS, upstream activating sequence.
(TIF)

S3 Fig Characterisation of induction of apoptosis using *hs-hid* and heat-shock of embryos. (a–c) Embryos ubiquitously expressing a caspase reporter and carrying the *hs-hid* transgene (*w;CyO hs-hid/+;da-GAL4*,*UAS-apoliner/+*) were heat-shocked for 0, 15, or 30 minutes and imaged from 60 minutes post heat shock. Relocalisation of nls-GFP (green) from membranes (via a CD8-RFP tether, purple) to the nucleus following caspase-dependent cleavage between these two fluorophores denotes cells in which caspases are active. Projections show ventral side of embryos at indicated times after heat-shock; arrows indicate examples of macrophages with RFP-positive ‘historical’ phagosomes; boxes show regions shown at higher magnification in (a’–c’). (d) Quantification of rate of generation of caspase-positive cells (cumulative numbers of GFP-positive nuclei over time) following heat-shock (shows mean of *n* = 3 per condition ± standard error of the mean). (e) Scatterplot showing numbers of macrophages per field of view on the ventral midline immediately prior to wounding in control and *hs-hid* embryos (*n* = 12 and 14, respectively; *P* = 0.995, Student’s *t* test); this prewound data corresponds to wounded embryo data set shown in Fig 5. (f–g) Scatterplots of data from control experiments to address whether genetic background, rather than induction of apoptosis, accounted for the impairment of wound responses seen in Fig 5: stage 15 *w;srp-GAL4*,*UAS-GFP/+*, *w;srp-GAL4*,*UAS-GFP/CyO* and *w;srp-GAL4*,*UAS-GFP/CyO hs-hid* embryos were wounded without heat-shock treatment. There was no difference in numbers of macrophages on the ventral side of the embryo prior to wounding (*n* = 14, 16, 14, respectively, g) or wound responses between genotypes (*n* = 11, 15, 13, respectively, f), indicating that neither the presence of a balancer chromosome (*CyO*) nor the *hs-hid* transgenic insertion affected either developmental dispersal or recruitment to wounds. Statistical analysis via one-way ANOVA with Tukey’s multiple comparison test (f-g); *P* values for (f–g) are as follows: *srp>GFP/+* versus *srp>GFP/CyO P* = 0.968 (f); *P* = 0.947 (g); *srp>GFP/+* versus *srp > GFP/CyO hs-hid P* = 0.987 (f); *P* = 0.869 (g); *srp > GFP/CyO* versus *srp > GFP/CyO hs-hid P* = 0.915 (f); *P* = 0.659 (g). Lines and error bars show mean and standard deviation in scatterplots; scale bars represent 20 μm in image panels. All data used to plot graphs may be found in Supporting information file S1 Data. GFP, green fluorescent protein; RFP, red fluorescent protein; UAS, upstream activating sequence.
(TIF)

S4 Fig The ability of macrophages to migrate is relatively unperturbed in *simu* mutants, such that wound responses are dominated by interactions with apoptotic cells. (a–b) Macrophages (mCherry, purple in merge) and apoptotic cells (GC3ai, green in merge) in controls (a) and *simu* mutants (b) on the ventral side of the embryo at stage 15. (b–b’) Show *simu* phenotypes ranging from severe (large amounts of uncleared GC3ai punctae, b) to mild (b’); some display a polarised localisation of GC3ai punctae (see S11 Movie and Fig 7c); even within mild examples persisting clusters of GC3ai punctae can be observed (asterisks, b’). (c–d) Quantification of speeds to wounds per macrophage (c), or expressed as the proportion migrating less than/greater than 2.2 μm/min (d). (e–f) Quantification of directionalities to wounds per macrophage (e), or expressed as the proportion exhibiting a directionality less than/greater than 0.8 (f); *P* < 0.0001 (c-d), *P* = 0.0004 (e) and *P* = 0.024 (f); *n* = 337 cells/19 embryos and 79 cells/21 embryos (c–f), Mann–Whitney test (c, e) or Fisher’s exact test (d, f). (g) Average time taken to repolarise toward wound for macrophages that respond to wounds in control and *simu* mutant embryos; *P* = 0.0642, *n* = 77 cells/11 embryos and 59 cells/12 embryos, respectively, Mann–Whitney test. Only macrophages that reached the wound were analysed (c–g). Genotypes are *w;;srp-3x-mCherry/da-GAL4,UAS-GC3ai* (control, a), *w;simu2;srp-3x-mCherry/da-GAL4,UAS-GC3ai* (*simu*, b-b’), *w;;crq-GAL4*,*UAS-GFP* (control, c–f), *w;simu*^*2*^;*crq-GAL4*,*UAS-GFP* (*simu*, c-f), *w;srp-GAL4*,*UAS-red stinger*,*Ecad-mCherry/+;crq-GAL4*,*UAS-GFP/+* (control, g), *w;simu*^*2*^,*srp-GAL4*,*UAS-red stinger*,*Ecad-mCherry/simu*^*2*^;*crq-GAL4*,*UAS-GFP/+* (*simu*, g); graphs show mean and standard deviation; *, ***, **** denote *P* < 0.05, *P* < 0.001, and *P* < 0.0001, respectively; scale bars represent 20 μm (a–b). All data used to plot graphs may be found in Supporting information file S1 Data. GFP, green fluorescent protein; UAS, upstream activating sequence.
(TIF)

S5 Fig Consequences of the loss of *simu* function to inflammation and wound healing. (a) Percentages of control (*w;;crq-GAL4*,*UAS-GFP*) and *simu* mutant embryos (*w;simu*^*2*^;*crq-GAL4*,*UAS-GFP*) that hatch to larvae following wounding; unwounded embryos mounted on slides but not wounded as a control. Ten embryos mounted per condition, experiment repeated in triplicate; no significant difference between control and *simu* for wounded or nonwounded hatching (ns; *P* > 0.99 and *P* = 0.748, respectively) via Fisher’s exact test. (b) Line graph showing percentage of embryos hatching at a given time post wounding/mock wounding per genotype. (c–d) Merged images showing TRE-GFP reporter (JNK activity, green) and red stinger-labelled macrophage nuclei (purple) immediately prior to wounding and 90 minutes post wounding in controls (*w;TRE-GFP;crq-GAL4*,*UAS-red stinger*, c) and *simu* mutant embryos (*w;TRE-GFP*,*simu*^*2*^;*crq-GAL4*,*UAS-red stinger*, d). (e) Numbers of macrophages at wounds over a 3-hour period post wounding of control (*w;srp-GAL4*,*UAS-red stinger*,*Ecad-mCherry/+;crq-GAL4*,*UAS-GFP/+*, *n* = 16) and *simu* mutant embryos (*w;simu*^*2*^,*srp-GAL4*,*UAS-red stinger*,*Ecad-mCherry/simu*^*2*^;*crq-GAL4*,*UAS-GFP/+*, *n* = 17); comparisons via Mann–Whitney tests. (f) Change in wound area in the 3 hours post wounding of controls and *simu* mutants normalised according to the wound size at 0 minutes for each embryo; wound areas measured from Ecad-mCherry channel. (f’–f”) Scatterplots of wound areas at 20 minutes and 3 hours post wounding (*P* = 0.61 and *P* = 0.0054, respectively, Mann–Whitney tests). Lines and error bars show mean and standard deviation (a, f’–f”) or SEM (b, e–f); *, **, ***, ns denote *P* < 0.05, *P* < 0.01, *P* < 0.001, and not significant; scale bars represent 20 μm, asterisks show centre of wounds (c–d). All data used to plot graphs may be found in Supporting information file S1 Data. GFP, green fluorescent protein; JNK, c-Jun N-terminal kinase; UAS, upstream activating sequence.
(TIF)

S6 Fig Laser-induced injury results in presentation of phosphatidylserine and membrane rupture with large amounts of nonapoptotic debris persisting at wound sites. (a–b) PBS (a) or PI injected (b) stage 15 embryos with GFP-labelled macrophages (*w;;crq-GAL4*,*UAS-GFP*) were imaged before and immediately after wounding, showing a dramatic accumulation of PI staining at the wound site immediately after laser-mediated ablation. Timings refer to exposure duration during image capture—a shorter exposure time (5 ms) reveals detail of nuclear localisation of PI, suggestive of necrotic cell death (b”); zoomed panel (b”’) shows close up of wound region in (b”). (c) As a positive control, embryos of the same genotype were heat treated for 15 minutes at 60 °C, resulting in widespread entry of PI in to cells and leakage of GFP from macrophages. (d–d’) Injection of texas red-labelled annexin V (as a marker for PS) into the vitelline space of stage 15 embryos with GFP-labelled macrophages (*w;;crq-GAL4*,*UAS-GFP*) reveals a rapid and sustained accumulation of annexin V at the wound edge and within the wound, including on cells in the process of being engulfed by those macrophages that have migrated to that site. Scale bars represent 20 μm (a–c) and 10 μm (d). GFP, green fluorescent protein; PBS, phosphate-buffered saline; PI, propidium iodide; PS, phosphatidylserine; UAS, upstream activating sequence.
(TIF)

S1 Table Fly genotypes used in this study. (TIF)

S1 Methods Methods exclusively used in supplementary figures. (DOCX)

S1 Movie Developmental dispersal of macrophages along the ventral midline in control and *simu* mutant embryos. GFP-labelled macrophages migrating on the ventral midline from late stage 12 onwards in a control (*w;;crq-GAL4*,*UAS-GFP*) and a *simu* mutant embryo (*w;simu*^*2*^;*crq-GAL4*,*UAS-GFP*). Macrophage dispersal is similar to controls, but on average there are reduced numbers present in this region. Scale bar represents 20 μm. GFP, green fluorescent protein; UAS, upstream activating sequence.
(AVI)

S2 Movie Developmental dispersal of macrophages in control and *simu* mutant embryos. Lightsheet movies of laterally-orientated control (*w;;crq-GAL4*,*UAS-red stinger*, upper embryo) and a *simu* mutant embryos (*w;simu*^*2*^;*crq-GAL4*,*UAS-red stinger*, lower embryo) containing red stinger-labelled macrophages undergoing developmental dispersal from stage 12 onwards. Macrophage dispersal is grossly similar to controls in *simu* mutants. Ant, anterior; post, posterior; hm, head mesoderm; vnc, ventral nerve cord; as, amnioserosa; gb, germ band; vm, vitelline membrane (outlined in green); both embryos are in the same orientation; scale bar represents 50 μm. UAS, upstream activating sequence.
(AVI)

S3 Movie Developmental dispersal of macrophages in control and *simu* mutant embryos in relation to apoptotic events. One hundred eighty-minute movies of mCherry-labelled macrophages (purple) and Caspase-ON reporter-labelled apoptotic cells (GC3ai, green) in a control (*w;;da-GAL4*,*UAS-GC3ai/srp-3x-mCherry*) and a *simu* mutant embryo (*w;simu*^*2*^;*da-GAL4*,*UAS-GC3ai/srp-3x-mCherry*) from early stage 12 onwards. Ventral views show macrophages as they migrate down the midline; embryos orientated diagonally across the field of view in order to image a greater proportion of the embryo. Note large numbers of GC3ai particles in *simu* mutant movie compared to the control and the slower migration speeds of macrophages in the *simu* mutant. Scale bars represent 20 μm. UAS, upstream activating sequence.
(AVI)

S4 Movie Macrophages migrate at slower speeds in *simu* mutants compared to controls. 30-minute movies of GFP-labelled macrophages at stage 15 on the ventral midline showing their random migration in control (*w;;crq-GAL4*,*UAS-GFP*, upper embryo) and *simu* mutant embryos (*w;simu*^*2*^;*crq-GAL4*,*UAS-GFP*, lower embryo). Movies repeat with second movie showing tracks of macrophage migration. Scale bar represents 20 μm. GFP, green fluorescent protein; UAS, upstream activating sequence.
(AVI)

S5 Movie Macrophage responses to wounds are perturbed in *simu* mutant embryos. Movies of the inflammatory migration of GFP-labelled macrophages to epithelial wounds in control (*w;;crq-GAL4*,*UAS-GFP*, embryo on left) and *simu* mutant embryos (*w;simu*^*2*^;*crq-GAL4*,*UAS-GFP*, embryo on right) at stage 15 of development on the ventral midline. Embryos wounded in the centre of the field of view. Scale bars represent 25 μm. GFP, green fluorescent protein; UAS, upstream activating sequence.
(AVI)

S6 Movie Induction of apoptosis visualised using the apoliner caspase reporter. Movies of *w;CyO hs-hid/+;da-GAL4*,*UAS-apoliner/+* embryos heat-shocked for 0 minutes (negative control), 5 minutes, 15 minutes, 30 minutes, and 60 minutes; timelapse movies begin 1-hour post heat shock. Caspase activity results in cleavage of apoliner, enabling its nls-GFP to translocate to the nucleus, whereas CD8-RFP remains on membranes. Caspase activity increases rapidly from 1-hour post heat shock, with the majority of caspase-positive cells remaining in an intact epithelium beyond the period of time in which inflammatory responses are typically followed. Movie shows green channel in first instance; second cycle shows merged image with GFP in green and RFP in purple. Scale bars represent 50 μm. GFP, green fluorescent protein; RFP, red fluorescent protein; UAS, upstream activating sequence.
(AVI)

S7 Movie Exogenous apoptosis impairs inflammatory responses of macrophages. Movies of GFP-labelled macrophage migration to wounds in a control embryo (*w;srp-GAL4*,*UAS-GFP/CyO*) and an embryo in which exogenous apoptosis has been introduced through *hid* expression (*w;srp-GAL4*,*UAS-GFP/CyO hs-hid*). Both embryos were heat-shocked for 15 minutes, with laser-wounding taking place 1-hour post heat-shock at a time-point when caspase activity is dramatically increasing, but when engulfment of cells destined to die has yet to take place. Fewer macrophages migrate to wounds in the presence of apoptotic cells, suggesting that apoptosis, but not necessarily phagocytosis of dying cells, can dampen inflammatory responses. Dotted lines show wound sites; scale bars represent 20 μm. GFP, green fluorescent protein; UAS, upstream activating sequence.
(AVI)

S8 Movie Rescue of wandering migration in *simu* mutants with no apoptosis. Hour-long movies of GFP-labelled macrophages in control (*w;;crq-GAL4*,*UAS-GFP*), apoptosis-null *Df(3L)H99* mutant (*w;;Df(3L)H99*,*crq-GAL4*,*UAS-GFP*), *simu* mutant (*w;simu*^*2*^;*crq-GAL4*,*UAS-GFP*) and apoptosis-null *simu* mutant embryos (*w;simu*^*2*^;*Df(3L)H99*,*crq-GAL4*,*UAS-GFP*) at stage 15. Removal of apoptotic cell death rescues macrophage speeds in a *simu* mutant background. Note the absence of vacuoles in *Df(3L)H99* and *simu*^*2*^;*Df(3L)H99* embryos due to the lack of apoptotic cell death. Scale bars represent 20μm. GFP, green fluorescent protein; UAS, upstream activating sequence.
(AVI)

S9 Movie Rescued initial responses to wounds in the absence of apoptosis in *simu* mutants. Hour-long movies of GFP-labelled macrophages in control (*w;;crq-GAL4*,*UAS-GFP*), apoptosis-null *Df(3L)H99* mutant (*w;;Df(3L)H99*,*crq-GAL4*,*UAS-GFP*), *simu* mutant (*w;simu*^*2*^;*crq-GAL4*,*UAS-GFP*) and apoptosis-null *simu* mutant embryos (*w;simu*^*2*^;*Df(3L)H99*,*crq-GAL4*,*UAS-GFP*) at stage 15 following wounding; initial image shows prewound image. Removal of apoptotic cell death rescues the percentage of macrophages responding to wounds in a *simu* mutant background. Note the absence of vacuoles in *Df(3L)H99* and *simu*^*2*^;*Df(3L)H99* embryos due to the lack of apoptotic cell death. Scale bars represent 20 μm. GFP, green fluorescent protein; UAS, upstream activating sequence.
(AVI)

S10 Movie Macrophage responses to injury in the presence of wild-type levels of uncleared apoptotic cell death. Ventral view of a stage 15 control embryo (*w;;srp-3x-mCherry/da-GAL4*,*UAS-GC3ai*) wounded at the midline showing robust macrophage (mCherry, purple) migratory responses to injury. Tracks of migration routes are overlaid; asterisk indicates position of wound. Very few apoptotic cell fragments (GC3ai, green) are visible, though occasionally interactions may be seen with GC3ai-labelled particles (boxed region); first half of movie shows overall response, before showing zooms of the cells from the boxed region in the prewound image. Scale bars represent 20 μm. UAS, upstream activating sequence.
(AVI)

S11 Movie Nonresponding macrophages interact with caspase-positive cell fragments impairing migration to wounds in *simu* mutants. Ventral view of a stage 15 *simu* mutant (*w;simu*^*2*^;*srp-3x-mCherry/da-GAL4*,*UAS-GC3ai*) wounded at the midline showing a weak macrophage (mCherry, purple) migratory response to injury. Tracks of migration routes are overlaid; asterisk indicates position of wound. The caspase reporter GC3ai (green) highlights numerous uncleared apoptotic fragments within the embryo and interactions between these and macrophages are associated with failed migration to the wound site (e.g., boxed region). First half of movie shows overall response, before showing zooms of the cells from the boxed region in the prewound image. Scale bars represent 20 μm. UAS, upstream activating sequence.
(AVI)

S12 Movie Enhanced macrophage egress from wounds in the absence of *simu*. Movies of GFP-labelled macrophages during wound responses to laser-induced epithelial wounds on the ventral midline in a control embryo (*w;;crq-GAL4*,*UAS-GFP*; embryo on left) and *simu* mutant that lacks apoptosis (*w;simu*^*2*^;*Df(3L)H99*,*crq-GAL4*,*UAS-GFP*; embryo on right). First repeat of wound response shows GFP channel only; second repetition shows all tracks of those cells present at 0 minutes post wounding superimposed over GFP channel; third repetition shows tracks of macrophages that leave the wound (1 cell in control embryo, 5 cells in the *simu*^*2*^;*Df(3L)H99* embryo). Scale bar represents 20 μm. GFP, green fluorescent protein; UAS, upstream activating sequence.
(AVI)

S13 Movie Macrophages interact with annexin V-labelled debris at wounds. Movie showing accumulation of annexin V to label exposed PS at wounds on the ventral surface during an inflammatory response in a control embryo (*w;;crq-GAL4*,*UAS-GFP*). Initial run of timelapse movie shows texas red-labelled annexin V alone, second repeat shows GFP-labelled macrophages (green) and annexin V (purple). Injection of fluorescent annexin V into the vitelline space labels the wound edge, suggesting that PS decorates stressed or damaged cells at these sites. Scale bar represents 20 μm. GFP, green fluorescent protein; PS, phosphatidylserine; UAS, upstream activating sequence.
(AVI)

## Abbreviations

cDCP-1: cleaved DCP-1
CED-1: cell death abnormality gene 1
CNS: central nervous system
COPD: chronic obstructive pulmonary disease
DCP-1: death caspase 1
EMILIN: elastin microfibril interface-located protein
GFP: green fluorescent protein
JNK: c-Jun N-terminal kinase
MEGF10: multiple epithelial growth factor-like domains 10
PBS: phosphate-buffered saline
PDGF: platelet-derived growth factor
PI: propidium iodide
PI3P: phosphatidylinositol 3-phosphate
PS: phosphatidylserine
RFP: red fluorescent protein
RNAi: RNA interference
SCAR: suppressor of cyclic AMP receptor
Simu: Six-microns-under
TGFβ: transforming growth factor β
UAS: upstream activating sequence
VEGF: vascular endothelial growth factor
VNC: ventral nerve cord
WAVE: WASP-family verprolin homologous protein
