# Supplementary figures and images for: Simu-dependent clearance of dying cells regulates macrophage function and inflammation resolution
Source: PLoS Biol. 2019 May 14;17(5):e2006741. doi: 10.1371/journal.pbio.2006741 (PMC6516643; doi:10.1371/journal.pbio.2006741)

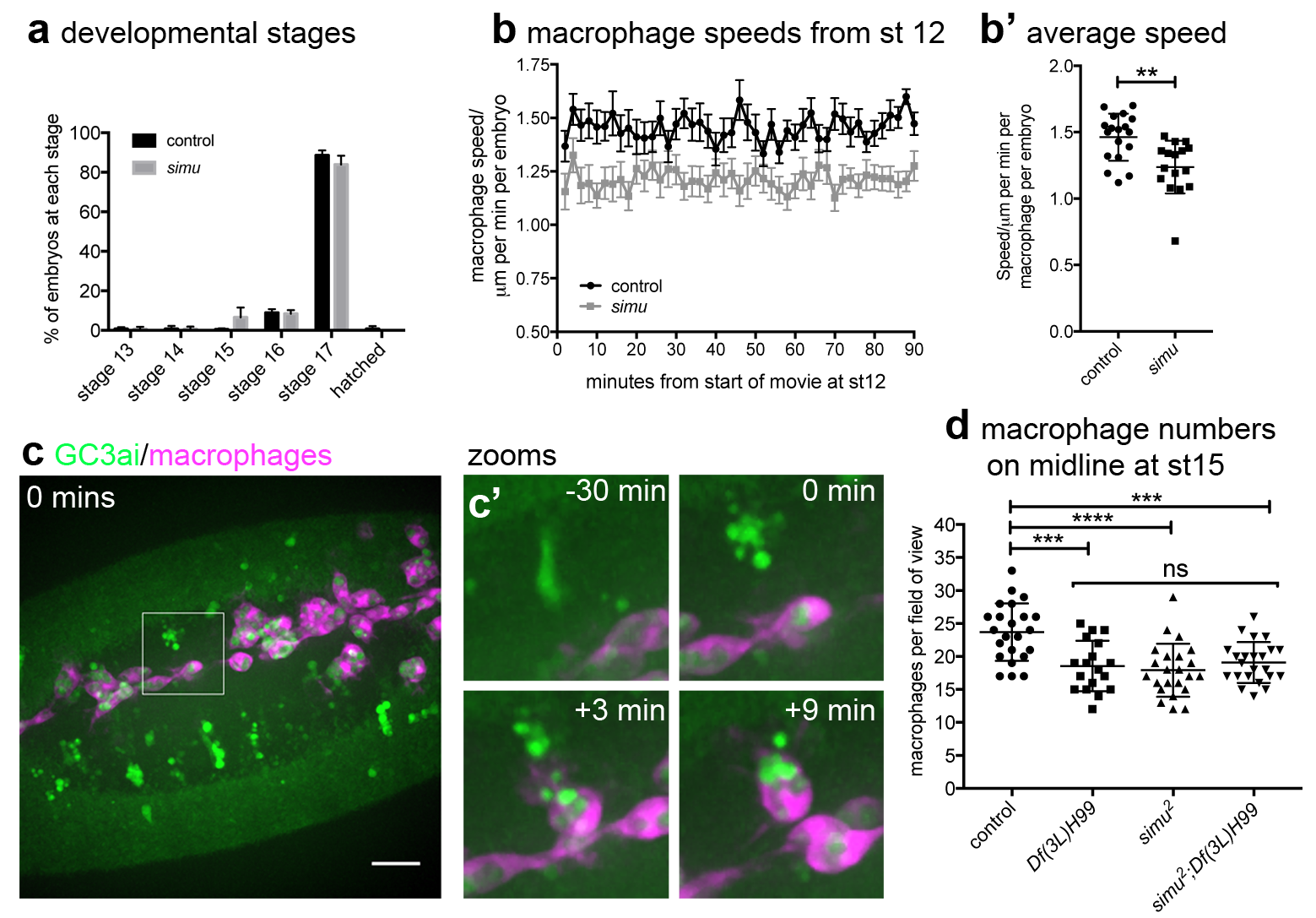

Supplement: S1 Fig — (a) Average percentages of control and simu mutant embryos at indicated developmental stages of following ageing for 28 hours at 18 °C (scored from 3 separate lays of 1.5 hours at 25 °C). No significant difference between proportions of embryos at stage 16 or earlier and stage 17 or later for these genotypes (Fisher’s exact test, P = 0.0581, n = 418 and 144 embryos, respectively). (b) Average speed per GFP-labelled macrophage, per embryo at each timepoint from late stage 12 onwards (when a single line of macrophages is present on the ventral midline) for 90 minutes in controls and simu mutant embryos (n = 18 and 16, respectively). (b’) Scatterplot of average speed per macrophage per embryo over 90 minutes from late stage 12. (c) Stills taken from a timelapse movie of srp-3x-mCherry-labelled macrophages dispersing along the ventral midline in a control embryo ubiquitously expressing a caspase-ON reporter (GC3ai). (c) Shows overall field of view; (c’) zooms showing onset of GC3ai fluorescence, fragmentation of the dying cell and its subsequent engulfment by a macrophage. (d) Numbers of macrophages on the ventral midline at stage 15 of development in controls (n = 23), Df(3L)H99 mutants (n = 18), simu mutants (n = 23) and simu;Df(3L)H99 double mutants (n = 23). Genotypes are w;;crq-GAL4,UAS-GFP (control) and w;simu2;crq-GAL4,UAS-GFP (simu) (a–b, d), w;;Df(3L)H99, crq-GAL4,UAS-GFP (Df(3L)H99) (d), w;simu2;Df(3L)H99,crq-GAL4,UAS-GFP (simu;Df(3L)H99) (d) and w;;da-GAL4,UAS-GC3ai/srp-3x-mCherry (c). Bars and data points show means, error bars show standard deviation (a, b’, d), or standard error of the mean (b); **, ***, and **** denote P < 0.01, P < 0.001, and P < 0.0001 via Mann–Whitney test (b’) or one-way ANOVA (d); scale bar represents 20 μm (c). All data used to plot graphs may be found in Supporting information file S1 Data. GFP, green fluorescent protein; UAS, upstream activating sequence. (TIF) [file pbio.2006741.s002.tif]

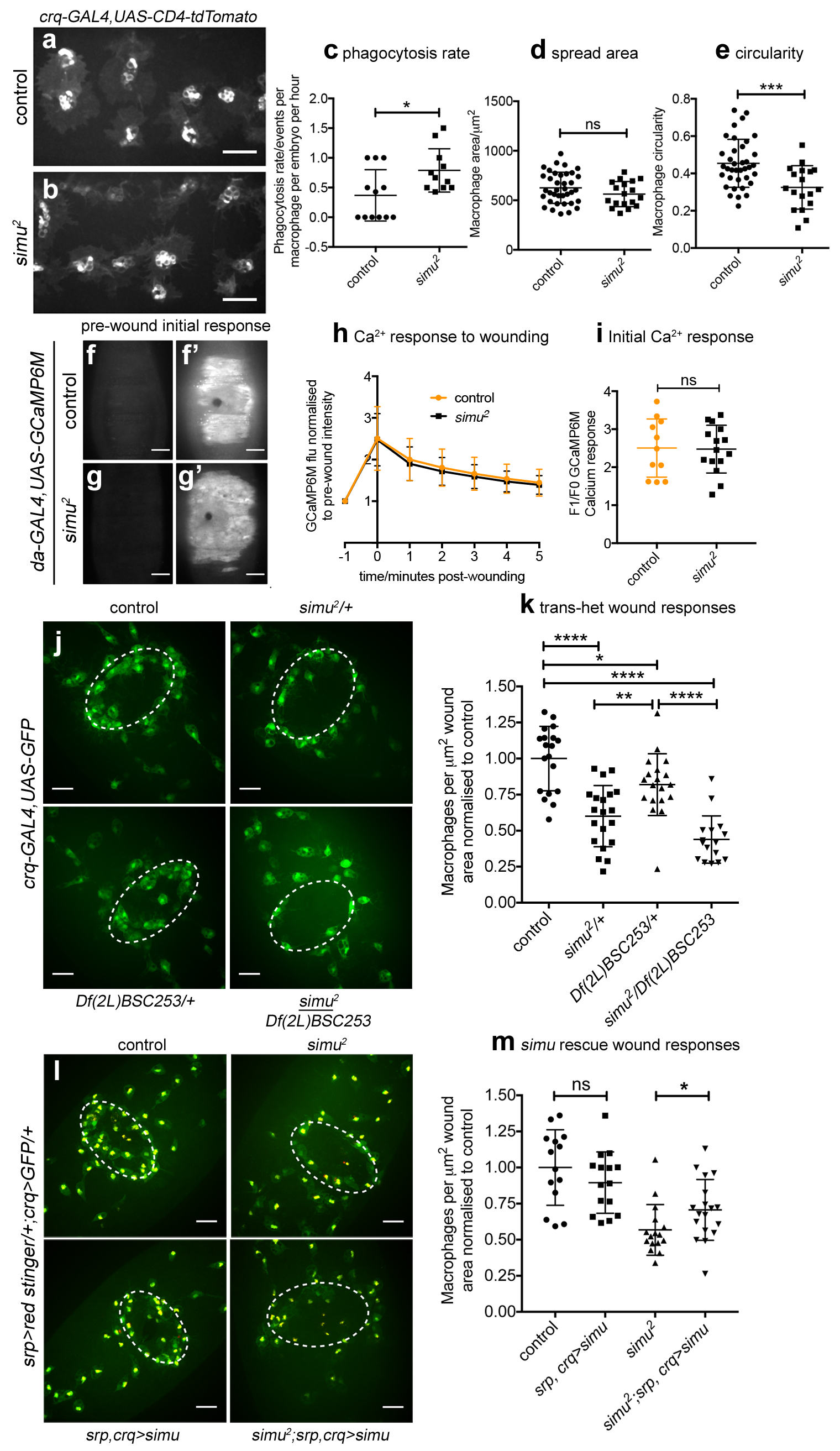

Supplement: S2 Fig — (a–b) Ventral images showing macrophage morphology in control (w;;crq-GAL4,UAS-CD4-tdTomato) and simu mutant embryos (w;simu2;crq-GAL4,UAS-CD4-tdTomato) at stage 15. (c–e) Scatterplots showing rates of phagocytosis (phagocytic events per hour, per macrophage, per embryo, c), macrophage spread area (μm2 per macrophage, d) and macrophage circularity (e). Statistical comparisons made via Mann–Whitney tests; n = 12 control and 11 simu2 embryos (c) and n = 37 control and 18 simu2 macrophages taken from 11 embryos (d–e). P = 0.028, 0.179, 0.0009 (c–e). (f–g) Images of cytoplasmic calcium levels (visualised using GCamP6M) in the epithelium prior to (f–g) and immediately after wounding (f’–g’) in control (w;;da-GAL4,UAS-GCamP6M, f-f’) and simu mutant embryos (w;simu2;da-GAL4,UAS-GCamP6M, g–g’). (h) Line graph showing timecourses of GCamP6M response (GCamP6M MGV of the entire embryonic field of view at each timepoint normalised to the prewound MGV) in control and simu mutant embryos (same genotypes as f–g). (i) Scatterplot of the ratio of initial response (F1) and prewound (F0) MGV in control and simu mutant embryos (n = 11 and 15, respectively; P = 0.80, Mann–Whitney test). (j) Ventral images showing wound responses in control (w;;crq-GAL4,UAS-GFP), simu/+ heterozygous (w;simu2/+;crq-GAL4,UAS-GFP), Df(2L)BSC253/+ heterozygous (w;Df(2L)BSC253/+;crq-GAL4,UAS-GFP) and simu/Df(2L)BSC253 trans-heterozygous embryos (w;simu2/Df(2L)BSC253;crq-GAL4,UAS-GFP) at 60 minutes post wounding. (k) Scatterplot showing wound responses corresponding to genotypes shown in (j). N numbers (left–right) are 19, 20, 19, 16; ns, *, **, ***, and **** denote not significant (P = 0.10), P = 0.043, 0.0079 and P < 0.0001, respectively, via one-way ANOVA with Dunn’s multiple comparisons post-test. (l) Ventral images showing wound responses (macrophages per wound area, normalised to control) at 60 minutes post wounding in controls (w;srp-GAL4,UAS-red stinger/+;crq-GAL4,UAS-GFP/+), simu mutants (w;simu2,srp- [file pbio.2006741.s003.tif]

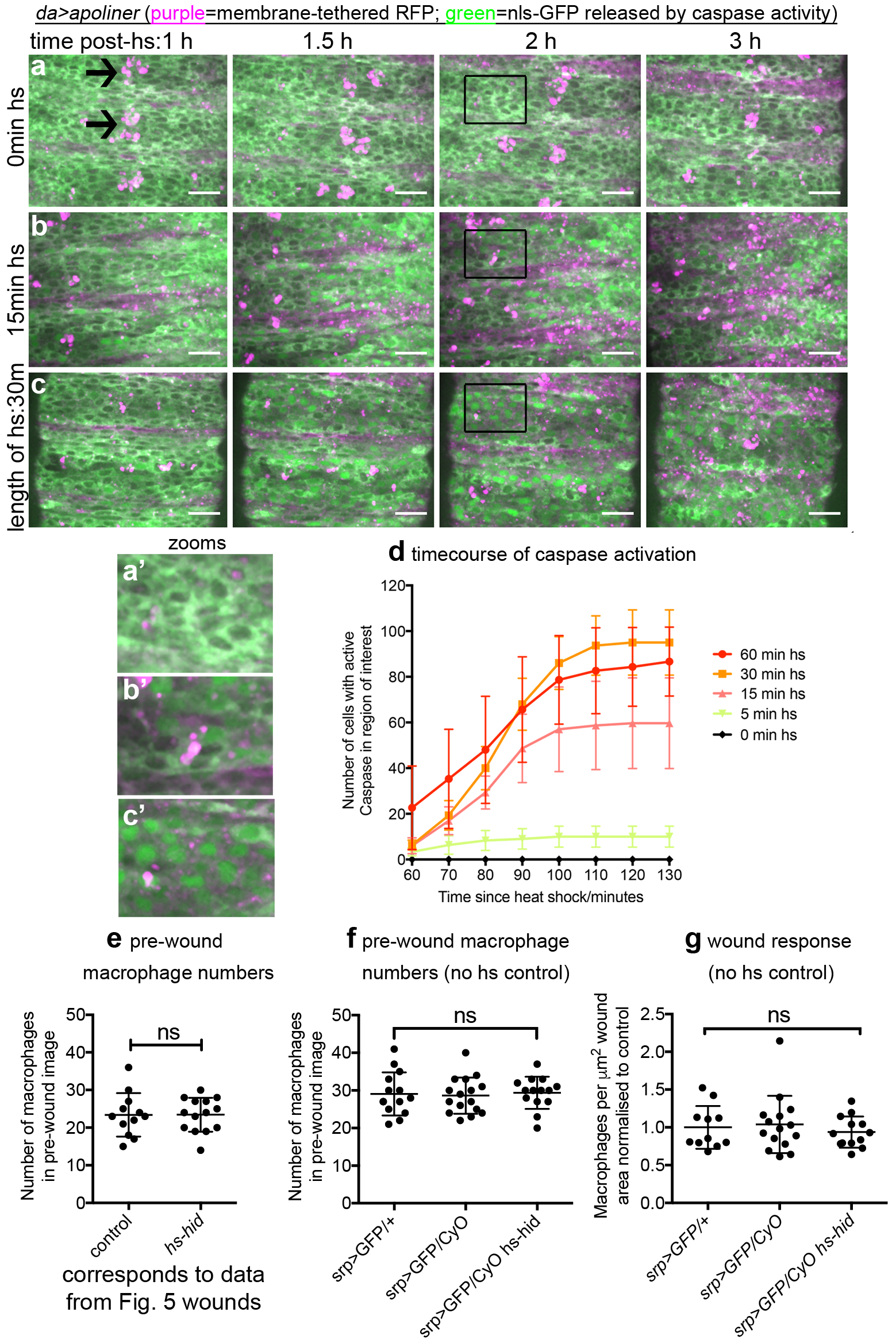

Supplement: S3 Fig — (a–c) Embryos ubiquitously expressing a caspase reporter and carrying the hs-hid transgene (w;CyO hs-hid/+;da-GAL4,UAS-apoliner/+) were heat-shocked for 0, 15, or 30 minutes and imaged from 60 minutes post heat shock. Relocalisation of nls-GFP (green) from membranes (via a CD8-RFP tether, purple) to the nucleus following caspase-dependent cleavage between these two fluorophores denotes cells in which caspases are active. Projections show ventral side of embryos at indicated times after heat-shock; arrows indicate examples of macrophages with RFP-positive ‘historical’ phagosomes; boxes show regions shown at higher magnification in (a’–c’). (d) Quantification of rate of generation of caspase-positive cells (cumulative numbers of GFP-positive nuclei over time) following heat-shock (shows mean of n = 3 per condition ± standard error of the mean). (e) Scatterplot showing numbers of macrophages per field of view on the ventral midline immediately prior to wounding in control and hs-hid embryos (n = 12 and 14, respectively; P = 0.995, Student’s t test); this prewound data corresponds to wounded embryo data set shown in Fig 5. (f–g) Scatterplots of data from control experiments to address whether genetic background, rather than induction of apoptosis, accounted for the impairment of wound responses seen in Fig 5: stage 15 w;srp-GAL4,UAS-GFP/+, w;srp-GAL4,UAS-GFP/CyO and w;srp-GAL4,UAS-GFP/CyO hs-hid embryos were wounded without heat-shock treatment. There was no difference in numbers of macrophages on the ventral side of the embryo prior to wounding (n = 14, 16, 14, respectively, g) or wound responses between genotypes (n = 11, 15, 13, respectively, f), indicating that neither the presence of a balancer chromosome (CyO) nor the hs-hid transgenic insertion affected either developmental dispersal or recruitment to wounds. Statistical analysis via one-way ANOVA with Tukey’s multiple comparison test (f-g); P values for (f–g) are as follows: srp>GFP/+ versus srp>GFP/CyO P = 0.96 [file pbio.2006741.s004.tif]

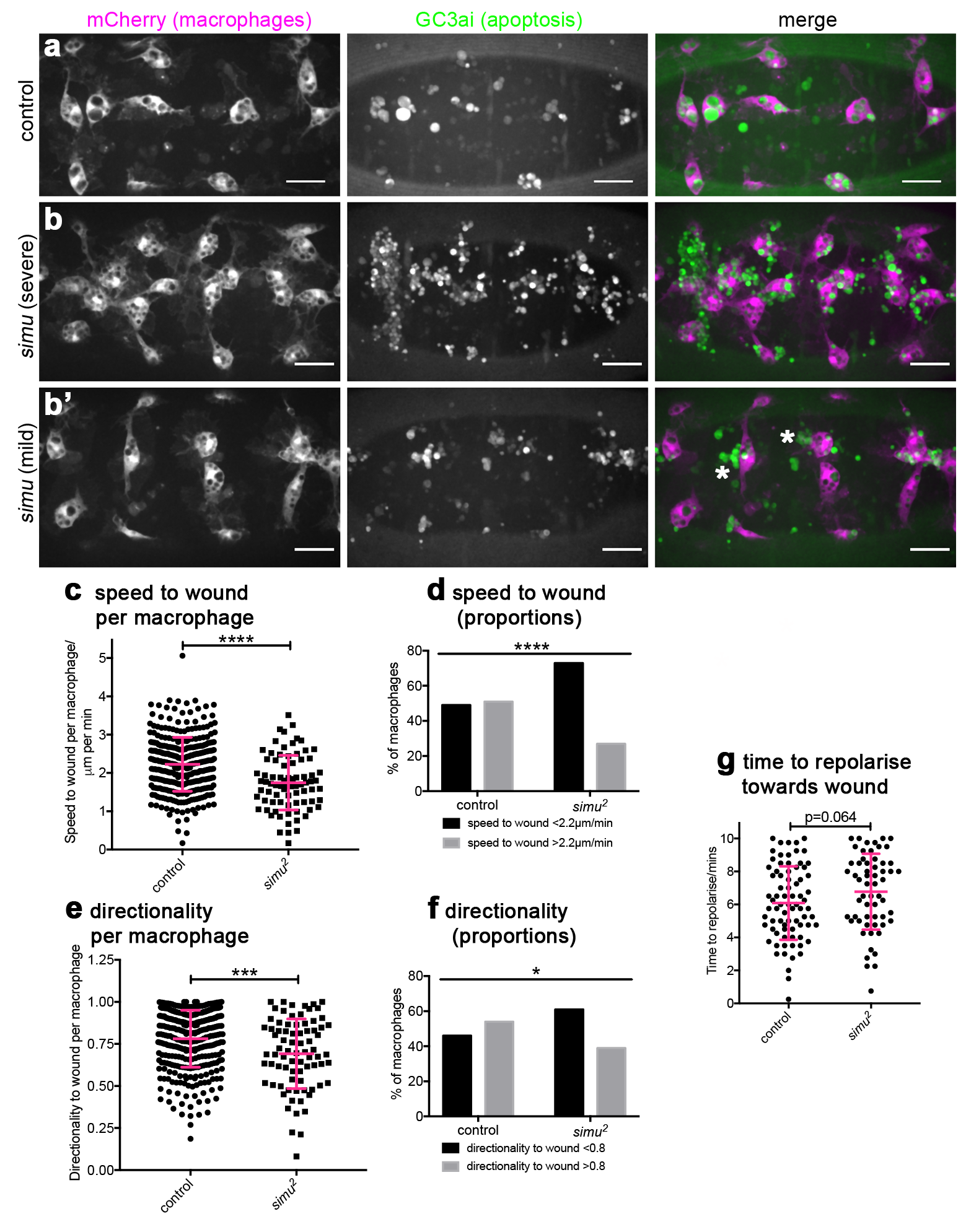

Supplement: S4 Fig — (a–b) Macrophages (mCherry, purple in merge) and apoptotic cells (GC3ai, green in merge) in controls (a) and simu mutants (b) on the ventral side of the embryo at stage 15. (b–b’) Show simu phenotypes ranging from severe (large amounts of uncleared GC3ai punctae, b) to mild (b’); some display a polarised localisation of GC3ai punctae (see S11 Movie and Fig 7c); even within mild examples persisting clusters of GC3ai punctae can be observed (asterisks, b’). (c–d) Quantification of speeds to wounds per macrophage (c), or expressed as the proportion migrating less than/greater than 2.2 μm/min (d). (e–f) Quantification of directionalities to wounds per macrophage (e), or expressed as the proportion exhibiting a directionality less than/greater than 0.8 (f); P < 0.0001 (c-d), P = 0.0004 (e) and P = 0.024 (f); n = 337 cells/19 embryos and 79 cells/21 embryos (c–f), Mann–Whitney test (c, e) or Fisher’s exact test (d, f). (g) Average time taken to repolarise toward wound for macrophages that respond to wounds in control and simu mutant embryos; P = 0.0642, n = 77 cells/11 embryos and 59 cells/12 embryos, respectively, Mann–Whitney test. Only macrophages that reached the wound were analysed (c–g). Genotypes are w;;srp-3x-mCherry/da-GAL4,UAS-GC3ai (control, a), w;simu2;srp-3x-mCherry/da-GAL4,UAS-GC3ai (simu, b-b’), w;;crq-GAL4,UAS-GFP (control, c–f), w;simu2;crq-GAL4,UAS-GFP (simu, c-f), w;srp-GAL4,UAS-red stinger,Ecad-mCherry/+;crq-GAL4,UAS-GFP/+ (control, g), w;simu2,srp-GAL4,UAS-red stinger,Ecad-mCherry/simu2;crq-GAL4,UAS-GFP/+ (simu, g); graphs show mean and standard deviation; *, ***, **** denote P < 0.05, P < 0.001, and P < 0.0001, respectively; scale bars represent 20 μm (a–b). All data used to plot graphs may be found in Supporting information file S1 Data. GFP, green fluorescent protein; UAS, upstream activating sequence. (TIF) [file pbio.2006741.s005.tif]

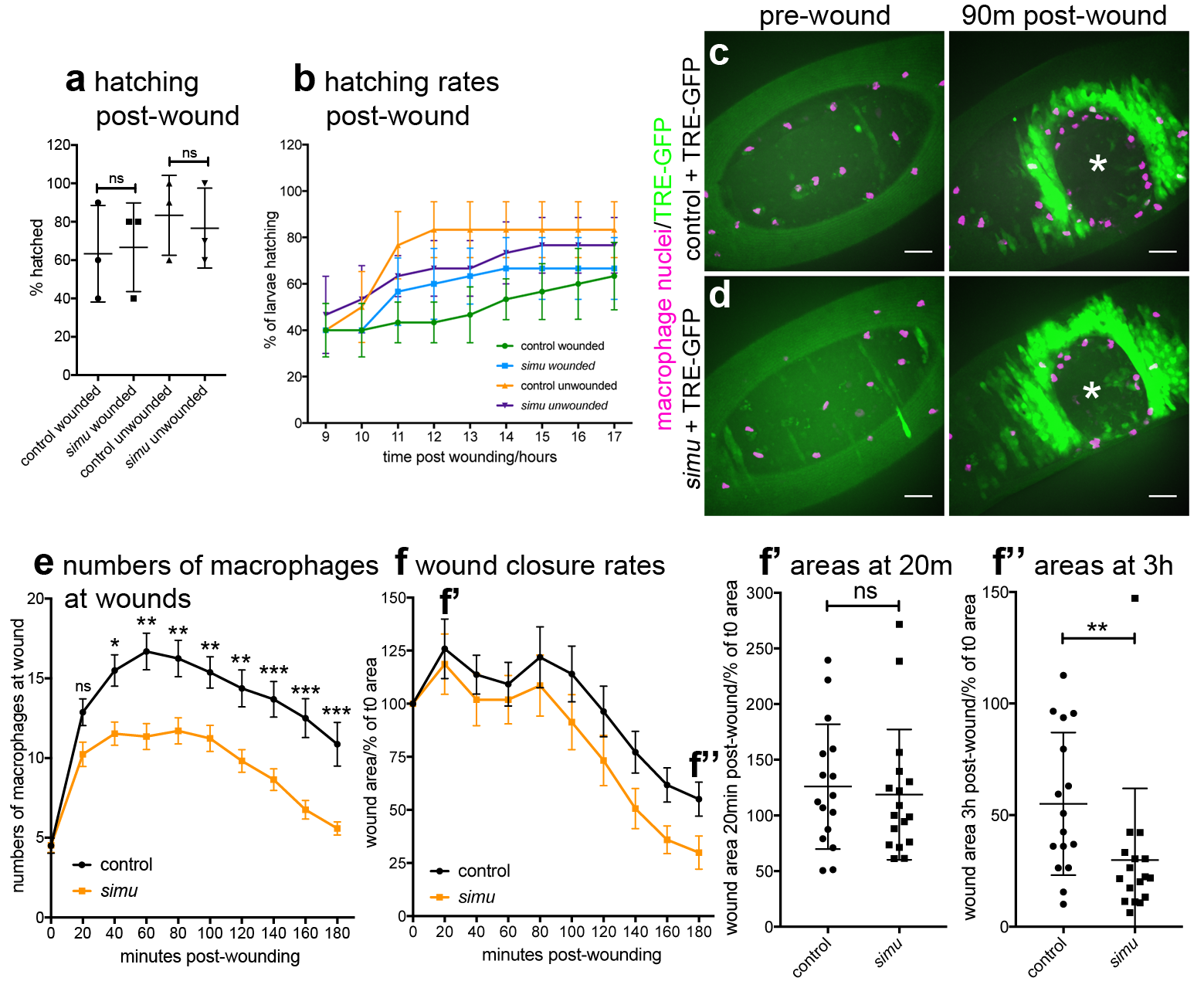

Supplement: S5 Fig — (a) Percentages of control (w;;crq-GAL4,UAS-GFP) and simu mutant embryos (w;simu2;crq-GAL4,UAS-GFP) that hatch to larvae following wounding; unwounded embryos mounted on slides but not wounded as a control. Ten embryos mounted per condition, experiment repeated in triplicate; no significant difference between control and simu for wounded or nonwounded hatching (ns; P > 0.99 and P = 0.748, respectively) via Fisher’s exact test. (b) Line graph showing percentage of embryos hatching at a given time post wounding/mock wounding per genotype. (c–d) Merged images showing TRE-GFP reporter (JNK activity, green) and red stinger-labelled macrophage nuclei (purple) immediately prior to wounding and 90 minutes post wounding in controls (w;TRE-GFP;crq-GAL4,UAS-red stinger, c) and simu mutant embryos (w;TRE-GFP,simu2;crq-GAL4,UAS-red stinger, d). (e) Numbers of macrophages at wounds over a 3-hour period post wounding of control (w;srp-GAL4,UAS-red stinger,Ecad-mCherry/+;crq-GAL4,UAS-GFP/+, n = 16) and simu mutant embryos (w;simu2,srp-GAL4,UAS-red stinger,Ecad-mCherry/simu2;crq-GAL4,UAS-GFP/+, n = 17); comparisons via Mann–Whitney tests. (f) Change in wound area in the 3 hours post wounding of controls and simu mutants normalised according to the wound size at 0 minutes for each embryo; wound areas measured from Ecad-mCherry channel. (f’–f”) Scatterplots of wound areas at 20 minutes and 3 hours post wounding (P = 0.61 and P = 0.0054, respectively, Mann–Whitney tests). Lines and error bars show mean and standard deviation (a, f’–f”) or SEM (b, e–f); *, **, ***, ns denote P < 0.05, P < 0.01, P < 0.001, and not significant; scale bars represent 20 μm, asterisks show centre of wounds (c–d). All data used to plot graphs may be found in Supporting information file S1 Data. GFP, green fluorescent protein; JNK, c-Jun N-terminal kinase; UAS, upstream activating sequence. (TIF) [file pbio.2006741.s006.tif]

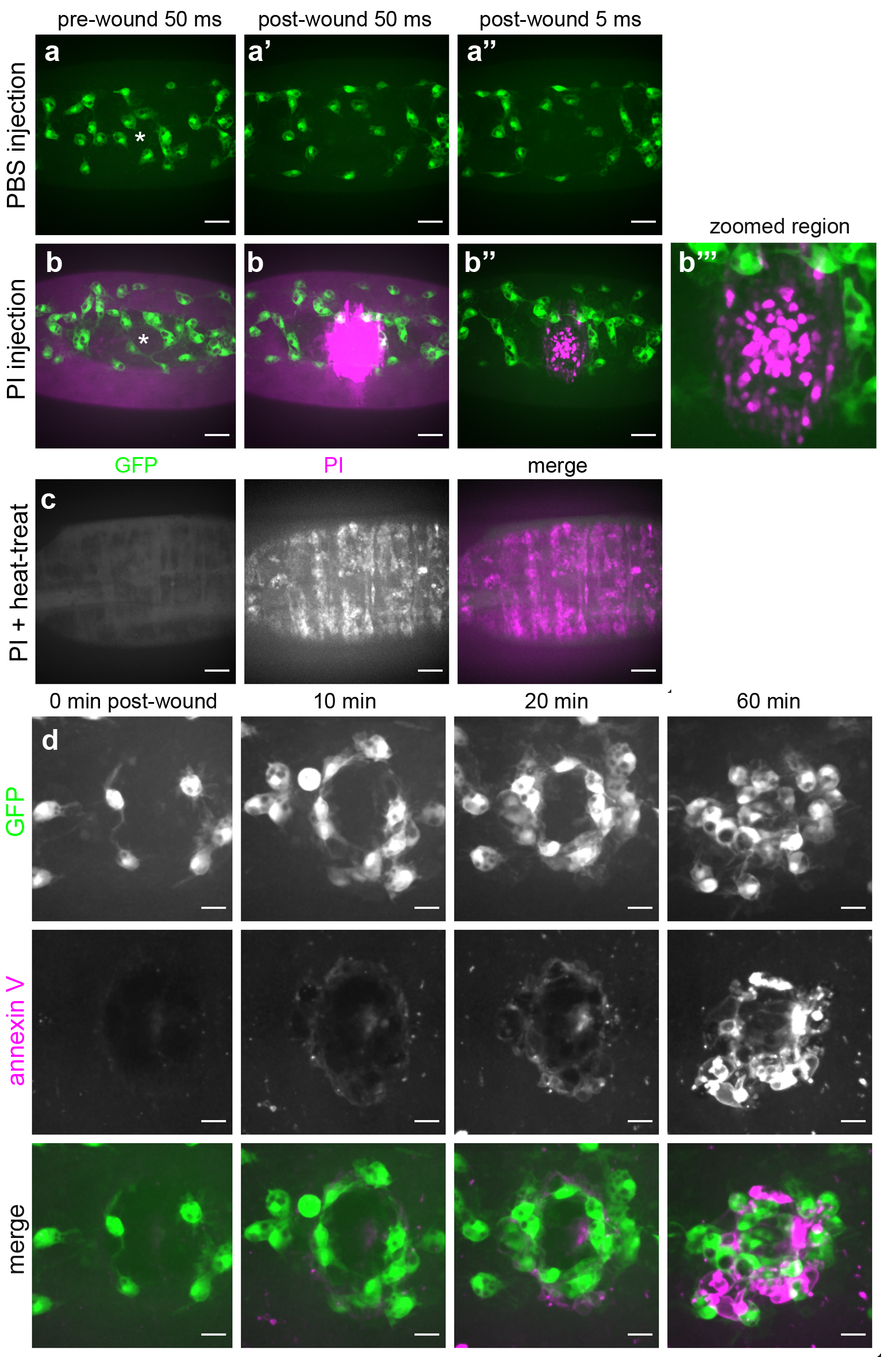

Supplement: S6 Fig — (a–b) PBS (a) or PI injected (b) stage 15 embryos with GFP-labelled macrophages (w;;crq-GAL4,UAS-GFP) were imaged before and immediately after wounding, showing a dramatic accumulation of PI staining at the wound site immediately after laser-mediated ablation. Timings refer to exposure duration during image capture—a shorter exposure time (5 ms) reveals detail of nuclear localisation of PI, suggestive of necrotic cell death (b”); zoomed panel (b”’) shows close up of wound region in (b”). (c) As a positive control, embryos of the same genotype were heat treated for 15 minutes at 60 °C, resulting in widespread entry of PI in to cells and leakage of GFP from macrophages. (d–d’) Injection of texas red-labelled annexin V (as a marker for PS) into the vitelline space of stage 15 embryos with GFP-labelled macrophages (w;;crq-GAL4,UAS-GFP) reveals a rapid and sustained accumulation of annexin V at the wound edge and within the wound, including on cells in the process of being engulfed by those macrophages that have migrated to that site. Scale bars represent 20 μm (a–c) and 10 μm (d). GFP, green fluorescent protein; PBS, phosphate-buffered saline; PI, propidium iodide; PS, phosphatidylserine; UAS, upstream activating sequence. (TIF) [file pbio.2006741.s007.tif]

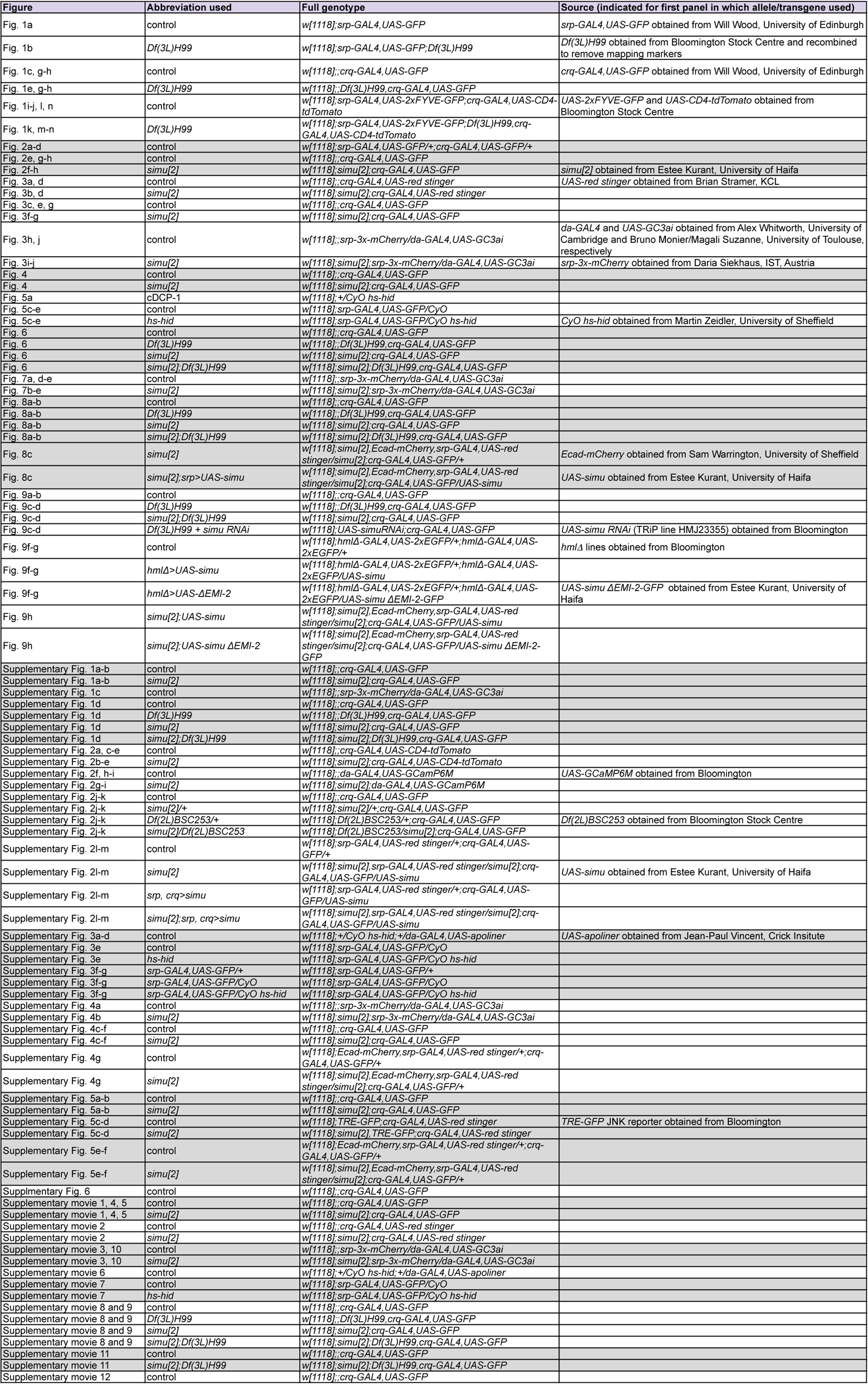

Supplement: S1 Table — (TIF) [file pbio.2006741.s008.tif]
