# Supplementary material for: Simu-dependent clearance of dying cells regulates macrophage function and inflammation resolution
Source: PLoS Biol. 2019 May 14;17(5):e2006741. doi: 10.1371/journal.pbio.2006741 (PMC6516643; doi:10.1371/journal.pbio.2006741)
Supplement: S1 Methods — (DOCX) [file pbio.2006741.s009.docx]

**S1 methods. Methods specific to the supplementary figures**

Lightsheet imaging of *Drosophila* embryos

Early stage 12 embryos were mounted vertically in 1% low-melting point agar (Sigma), within a glass capillary (outer diameter 1mm, inner diameter 0.68mm). Capillaries were submerged in PBS within a Zeiss Lightsheet microscope and imaged using a 20x Plan-Apochromat objective lens (NA 1). Samples were allowed to develop at 28^o^C and imaged using a 568nm laser. Stacks of 80µm and timelapse movies were taken every 15.1 seconds. Images were then re-sampled in the Zen Black software (Zeiss). Following this, files were opened in Fiji and a sub-stack created using the slice keeper tool to make stacks with time points every 90.6 seconds.

Analysis of developmental timings

To measure time taken to develop, flies were allowed to lay embryos on apple juice agar plates in laying cages for 1.5 hours at 25^o^C. Plates with embryos were then removed and aged at 18^o^C for a further 28 hours. Embryos were then washed off the apple juice plates and stages scored using an MZ205 fluorescent stereomicroscope (Leica). Embryos were scored in the following categories stage 12, stage 13, stage 14, stage 15, stage 16, stage 17, hatching to L1 larvae.

Quantification of macrophage morphology

Cell outlines were traced from maximum projections of macrophages on the ventral side of the embryo in which cell edges could easily be discerned and the whole cell was in focus. Fiji was used to calculate spread area and circularity (4π(area/perimeter^2^); a value of 1 indicates a perfect circle, with cells becoming increasingly elongated as their value apporaches 0. Measurements were made on blinded images.

Quantification of calcium responses

Maximum projections of the GCamP6M channel were generated for each timepoint; a depth of 20μm from where the epithelium first comes into focus was used per projection. GCamP6M fluorescence (mean gray value) was calculated for the entire region of the embryo in the field of view (demarcated by the vitelline membrane) and this value was normalised compared to the pre-wound value to measure changes in cytoplasmic calcium levels over time following wounding. All analysis was performed on blinded projections.

Analysis of macrophage chemotaxis to wounds

Macrophages that chemotaxed to wounds were tracked from their initial position at 0-minutes post-wounding or when they appeared in the field of view, until they reached the wound. 60-minute wounding movies were used for this analysis, using macrophages labeled via *crq-GAL4,UAS-GFP*. Macrophages that did not respond to wounds (defined as cells that did not reach the wound during the 60-minute timelapse movie) were not analysed. Tracking was performed using the manual tracking plugin and the chemotaxis tool was used to calculate average speed per cell and directionality (ratio of the shortest route possible/distance moved) in Fiji.

Responding macrophage populations were broken down into good/poor responders based upon the median of the control population (2.2μm per min and a directionality of 0.8) for contingency test analyses (Fisher’s exact test).

Movies were taken at a higher frame rate (every 15 seconds for 10 minutes) to analyse repolarisation towards wounds. Only macrophages that responded to wounds within this time period were scored and only those that were not obviously polarised towards the wound at 0-minutes post-wounding. Time taken to polarise towards the wound was manually scored from blinded movies of these wound responses. Macrophages were labeled using one copy each of *srp-GAL4,UAS-red stinger* and *crq-GAL4,UAS-GFP* (control genotype was *w;srp-GAL4,UAS-red stinger,Ecad-mCherry/+;crq-GAL4,UAS-GFP/+*), which was sufficient for clear enough labeling to accurately discern protrusions for 10 minutes of high frequency imaging using a spinning disk microscope.
